# Silencing of circRERE(4-5) inhibits ONECUT2-mediated tumorigenesis and metastasis in gastric cancer

**DOI:** 10.3389/fimmu.2026.1686702

**Published:** 2026-03-06

**Authors:** Hua Xiao, Bowen Yu, Min Ma, Chonglei Zhong, Ming Shi, Yongxin Ye, Qionggui Hu, Shiyi Liu, Qinpeng Long, Kaiyu Zhu, Fei Long

**Affiliations:** 1 Department of Hepatobiliary and Intestinal Surgery, The Affiliated Cancer Hospital of Xiangya School of Medicine, Central South University/Hunan Cancer Hospital, Changsha, Hunan, China; 2 Department of Gastrointestinal Surgery, The Third Xiangya Hospital of Central South University, Changsha, Hunan, China; 3 Department of Radiotherapy, Hunan Cancer Hospital/The Affiliated Cancer Hospital of Xiangya School of Medicine, Central South University, Changsha, Hunan, China; 4 Department of Thoracic Surgery, Ruijin Hospital, Shanghai Jiao Tong University School of Medicine, Shanghai, China; 5 Department of Pediatrics, The First Affiliated Hospital of University of South China, Hengyang, Hunan, China; 6 Center for Gastrointestinal Surgery, The First Affiliated Hospital, Sun Yat-Sen University, Guangzhou, Guangdong, China

**Keywords:** circRERE(4-5), circular RNA, gastric cancer, metastasis, ONECUT2

## Abstract

**Introduction:**

The leading cause of mortality for gastric cancer (GC) patients is metastasis. Investigating the mechanisms that drive the dissemination of GC could reveal crucial aspects of tumour biology and potentially lead to valuable therapeutic strategies. Circular RNAs (circRNAs), which are extensively expressed in tumours, are involved in a range of biological processes, such as cancer metastasis and cancer immunity. In the present study, the role of circRNAs in the progression and dissemination of GC was investigated.

**Methods:**

CircRNAs expression were analyzed using GEO datasets and qRT-PCR. The role of circRNAs in the progression of GC was investigated using functional assays, molecular experiments, and in vivo xenograft models.

**Results:**

We identified a circRNA, circRERE(4-5) (circBase ID: hsa_circ_0009594), which facilitated GC progression. CircRERE(4-5) was notably elevated in GC tissues and cells, and plasma circRERE(4-5) levels correlated closely with GC size and metastasis. Knockdown of circRERE(4-5) suppressed the growth and movement of GC cells through a pathway involving miR-571 and one cut homeobox 2 (ONECUT2). Moreover, antisense oligonucleotides targeting circRERE(4-5) suppressed the growth and spread of xenograft tumours in mice.

**Conclusion:**

Our research uncovers the functional and diagnostic significance of circRERE(4–5) and highlights circRNAs as pivotal factors in GC development and spread.

## Introduction

Gastric cancer (GC) continues to be one of the most common and deadly cancers globally, with over 960,000 new cases (accounting for 4.9% of all cancer types) and more than 650,000 fatalities (6.8% of all cancer-related deaths) each year (1). This high incidence of mortality is largely due to the aggressive nature of GC, which often metastasizes even from early-stage primary tumours. Metastatic spread is the leading determinant of unfavourable outcomes in GC patients, as approximately 90% of cancer-related deaths are attributed to metastatic disease (2–4). Understanding the mechanisms underlying metastasis could uncover new vulnerabilities in GC cells. However, the cellular and molecular processes driving GC metastasis remain not fully understood.

Circular RNAs (circRNAs) arise from precursor mRNAs through back-splicing that covalently joins a downstream 5′ splice site to an upstream 3′ splice site, forming either a lariat-driven or direct circularization product (5, 6). The resulting covalently closed loop lacks free termini, confers strong resistance to RNase-R digestion, and gives circRNAs an intracellular half-life markedly longer than that of linear RNAs (7, 8). Over the past decade, research into circRNA biology has uncovered their essential roles in tumorigenesis and cancer immunity (9, 10). Recent studies have indicated that circRNAs play vital roles in cancer metastasis through various mechanisms, such as acting as miRNA sponges, protein interactors, or even protein templates (11, 12).

In GC, individual circRNAs have been functionally linked to tumour initiation and progression. For instance, hsa_circ_0136666 stimulates GC progression and micro-environment formation by sponging miR-375-3p to up-regulate PRKDC, which phosphorylates and stabilises PD-L1, thereby suppressing CD8+ T-cell immunity (13). CircATP8A1 promotes GC growth and invasion by packaging into exosomes, sponging miR-1-3p and activating STAT6 to polarise macrophages toward an M2, tumour-promoting phenotype (14). Beyond miRNA sponging, several GC-related circRNAs serve as protein scaffolds, mRNA interactors, or even protein templates, underscoring their mechanistic versatility. For example, circURI1 (hsa_circ_0000921) restrains GC cell migration, invasion and metastasis by directly binding hnRNPM to reprogramme alternative splicing of motility-related genes (15). CircUSP1 (hsa_circ_000613) drives GC growth and metastasis by binding the RRM1 domain of HuR, blocking β-TrCP-mediated ubiquitination and stabilising the oncoprotein. This post-transcriptionally amplifies USP1 and Vimentin, which mediate the effects of circUSP1 (16). CircTFRC binds *SCD1* mRNA and recruits ELAVL1 (HuR) to boost SCD1 translation, thereby blocking lipid peroxidation, ferroptosis and driving GC progression (17). CircDIDO1 encodes a 529-aa protein that blocks PARP1 activity and drives RBX1-mediated PRDX2 degradation, thereby suppressing GC cell proliferation, migration and metastasis (18). Despite these advances, the spectrum of circRNAs that govern GC metastasis remains incompletely understood (19).

Here, we profiled circRNA expression in GC tissues and identified circRERE(4–5) (hsa_circ_0009594) as a prominently up-regulated transcript. Functional studies demonstrate that circRERE(4–5) sponges miR-571 to de-repress ONECUT2 (one cut homeobox 2), accelerating GC cell proliferation and motility. Therapeutic delivery of antisense oligonucleotides (ASOs) targeting circRERE(4–5) effectively suppressed tumour growth and metastasis in animal models, suggesting that blocking this circRNA may represent a novel anti-metastatic strategy for GC.

## Materials and methods

### Patients and samples

Primary GC tissues and matched normal adjacent tissues (situated more than 5 cm from the tumour) were obtained from 30 patients who underwent gastrectomy for GC without prior neoadjuvant therapy at The Third Xiangya Hospital of Central South University between January and December 2024. Peripheral blood specimens were collected from 24 GC patients and 12 healthy controls matched for age and sex. All samples were immediately cryopreserved using liquid nitrogen and stored at -80°C. This study was sanctioned by the Ethics Committee of The Third Xiangya Hospital of Central South University (Approval No: 2024-S187), and each patient provided signed informed consent documentation.

### Cell lines and cell culture

The human normal gastric mucosal epithelial cell line gastric epithelial cell line-1 (GES-1) and human GC cell lines adenocarcinoma gastric cell line (AGS), human gastric cancer cell line-27 (HGC-27), Mitsubishi-Kagaku Nihon University (MKN)-28 and MKN-45 were sourced from the Shanghai Institutes for Biological Sciences. GES-1 cells were maintained in DMEM (Gibco, USA), AGS cells in F-12K medium (Gibco, USA), and HGC-27, MKN-28 and MKN-45 cells in RPMI-1640 medium (Gibco, USA). Each culture medium was enriched with 10% FBS (Gibco, USA), 100 mg/mL streptomycin (Gibco, USA) and 100 IU/mL penicillin (Gibco, USA). The cells were maintained in a humidified incubator at 37°C with 5% CO_2_. The identity of the cell lines was confirmed by short tandem repeat analysis and they were also tested for isozyme profiles, cell viability and mycoplasma contamination.

### CircRNA microarray analysis

CircRNA microarray datasets GSE93541 (comprised of 3 plasma samples from GC patients and 3 from healthy individuals), GSE83521 (encompassing 6 GC and 6 adjacent normal mucosa tissues), and GSE194384 (featuring 3 matched GC and normal tissues) were retrieved from the GEO repository. CircRNA expression profiling in GC tissues was performed using the Arraystar Human CircRNA microarray V1 for GSE93541 and GSE83521, and V2 for GSE194384, on the GPL19978 and GPL21825 platforms, respectively. The V2 platform contains approximately 10,000 additional probe sets and updated back-splice junction annotations compared with V1; therefore, we used GSE194384 solely for validation of the top candidate (circRERE(4–5)) rather than for direct numerical integration with the discovery cohorts. Bioinformatics analysis was employed to pinpoint differentially expressed circRNAs between GC patients and healthy controls (*P* < 0.05), with a log_2_FC ≥ 1.5 cut-off used to identify significantly elevated circRNAs in GC tissues or plasma.

### Bioinformatics analysis

To investigate the expression levels of circRNAs in normal vs. tumour tissues, the GEO database was employed. For circRNA search and annotation, circBase and circBank databases were accessed. Potential circRNA–miRNA interactions were predicted using circBank and circInteractome. Additionally, TargetScan 8.0, miRDB and miRTarBase databases were utilized to predict the target mRNAs of miRNAs.

### Total RNA extraction and quantitative real-time PCR

Total RNA was extracted using Invitrogen TRIzol^®^ Reagent and subsequently reverse-transcribed into complementary DNA (cDNA) using the TOYOBO RiverTra Ace qPCR RT Master Mix with genomic DNA (gDNA) Scavenger. qRT–PCR was conducted on a Roche LightCycler 480 qRT–PCR System with the TOYOBO qPCR Mix Kit. The relative quantifications of circRNAs and mRNAs were determined by the 2^-ΔΔCt^ method, with glyceraldehyde-3-phosphate dehydrogenase (*GAPDH*) serving as the endogenous reference. The primer sequences are listed in **Supplementary Table 1**.

### RNase R treatment assays

In RNA stability assays, 2 μg of total RNA from AGS and HGC-27 cells was subjected to treatment with 5 U/μg RNase R at 37°C for 30 minutes before undergoing reverse transcription. Following this, the levels of circRERE(4–5), *RERE* mRNA and *GAPDH* mRNA were measured using qRT–PCR.

### Actinomycin D treatment assays

AGS and HGC-27 cells were seeded into 6-well plates and, once they reached about 60% confluence after 24 hours, were treated with either 5 μg/mL actinomycin D or DMSO (Sigma-Aldrich). Specimens were procured at designated intervals (0, 2, 4, 6, 8 and 12 hours) for qRT–PCR to evaluate relative expression levels of circRERE(4–5) and linear *RERE* mRNA, allowing for the determination of their respective half-lives.

### Nuclear and cytoplasmic extraction

The isolation of cytoplasmic and nuclear fractions was carried out using a Norgen Biotek Cytoplasmic & Nuclear RNA Purification Kit in accordance with the manufacturer’s instructions. Specifically, AGS or HGC-27 cells underwent lysis in chilled Lysis Buffer J for 5 minutes while kept on ice. The resulting mixtures were subjected to maximum-speed centrifugation for 3 minutes in a benchtop centrifuge, where the supernatant yielded the cytoplasmic portion and the precipitate contained the nuclear fraction. Cytoplasmic and nuclear RNAs were subsequently bound to a column, washed with Wash Solution A and then purified.

### ShRNAs, plasmid construction, lentivirus, and cell transfection

GeneChem designed three short hairpin RNAs (shRNAs) that targeted the back-splice junction (BSJ) regions of circRERE(4–5) (sh-circRERE) and also provided a control shRNA (sh-NC). The full-length sequence of circRERE(4–5) was amplified and cloned into a circRNA-specific overexpression lentiviral vector, GV689 (GeneChem), which contained two homology arms upstream and downstream of the circRNA sequence to promote circRNA cyclization. Additionally, the overexpression vectors (GV367) for *ONECUT2* were obtained from GeneChem. The transfection of GC cells with these shRNAs or vectors was performed utilizing Lipofectamine 3000 (Invitrogen) following the supplier’s protocols. For stable cell line establishment, lentiviruses containing shRNAs targeting circRERE(4–5) BSJ sites, and carrying green fluorescent protein and puromycin resistance genes, were purchased from GeneChem. Infected cells underwent exposure to 5-10 μg/mL puromycin (Gibco) after 72 hours to select for circRERE(4–5) knockdown. The efficiency of knockdown was confirmed using qRT–PCR. The target sequence for sh-circRERE(4–5) was: GGCCTGTAGGGACTGTGTGTA.

### CCK-8 assay

Cell viability was evaluated using the Cell Counting Kit-8 (CCK-8) assay. GC cells were plated into 96-well plates at a density of 2,000 cells per well and maintained for 0, 24, 48 or 72 hours. Subsequently, 10 µL of CCK-8 solution (Abbkine) was introduced into each well and incubated for 1.5 hour. The optical density at 450 nm was measured using a BioTek microplate reader (model ELX800, BioTek Instruments).

### Plate colony formation assay

Colony formation assays were performed to evaluate cell proliferation. Around 200–500 GC cells were seeded into each well of 6-well plates, with 3 replicates for each group. After 7–10 days of incubation, the cells were fixed with 4% paraformaldehyde for 40 minutes and then stained with 0.25% crystal violet for 20 minutes, followed by air drying at room temperature. Colonies containing ≥ 50 cells were photographed and quantified.

### Transwell assay

Transwell assays were used to evaluate cell migration with 24-well chambers (Corning) that had 8 μm pores. Approximately 2–6 × 10^4^ GC cells were resuspended in 150 μL of serum-free medium and placed into the upper chamber and 500 μL of complete medium into the lower chamber. Following 24-hour incubation, non-migratory cells on the upper membrane surface were eliminated and migratory cells on the lower surface were fixed with 4% paraformaldehyde for 40 minutes and then stained with 0.25% crystal violet for 20 minutes at 25°C. The quantity of migrated cells was determined by counting in 3 random fields observed under an Olympus BX51 inverted microscope at ×100 magnification.

### Wound healing assays

Approximately 6 × 10^5^ GC cells were seeded in 6-well plates. Once the GC cells had overgrown the plate bottom, scratches were made in each well using a pipette tip. Following the removal of cellular fragments, each wound was recorded at 0 hour using an inverted microscope (Olympus, Japan). Then, cells were continually cultured in basal medium. Twenty-four hours later, the wounds were imaged after the same washing procedure aforementioned. The rate of cell migration, also known as the wound healing rate, was determined using the formula: Wound Healing Rate (%) = [(Initial Mean Cell Distance - Mean Cell Distance at Time t)/Initial Mean Cell Distance] × 100%.

### Western blotting and antibodies

Cells were lysed on ice for 15 minutes using cell lysis buffer that included a protease inhibitor and a phosphatase inhibitor. The lysates were then centrifuged at 12,000 × g for 20 minutes at 4 °C. Protein concentrations in the supernatants were measured using a BCA assay (Beyotime Biotechnology). Protein samples (20 µg) were separated on 10% SDS-PAGE gels and transferred to PVDF membranes (Millipore) with 0.2 µm pores. The membranes were blocked in TBST with 8% skim milk for 1 hours and incubated with primary antibodies at 4 °C overnight. After washing, membranes were exposed to HRP-conjugated secondary antibodies for 1.5 hour at 25 °C, and signal development was performed using enhanced chemiluminescence (Thermo Fisher Scientific). Imaging and densitometric analysis were conducted using the ChemiDoc Touch Imaging System (Bio-Rad) and Image Lab Software. The antibodies used were anti-ONECUT2 (Proteintech, #21916-1-AP; 1:1,000), anti-AGO2 (Proteintech, # 67934-1-Ig; 1:1,000), anti-RERE (abcam, ab217756; 1:1,000), and anti-GAPDH (Proteintech, #10494–1-AP; 1:6,000).

### RNA-binding protein immunoprecipitation

RIP assays were conducted using the EZ-Magna RIP Kit (Merck, KGaA) in accordance with the manufacturer’s instructions (20). Immunoprecipitated RNAs were subsequently analysed by qRT–PCR and enrichment was calculated relative to input RNA.

### RNA pull-down assay

For RNA pull-down experiments, biotinylated probes specific to circRERE(4–5) and control sequences were designed and synthesized by Sangon Biotech. The pull-down procedure was carried out using the Pierce™ Magnetic RNA–Protein Pull-Down Kit (Pierce Biotechnology) in accordance with the manufacturer’s guidelines (20). The captured RNAs were subsequently isolated and analysed using qRT–PCR.

### Animal studies

Female BALB/c nude mice (4–5 weeks old, 18–20 g) and female NOD-SCID mice (4–5 weeks old, 18–20 g), were sourced from SLAC Laboratory Animal Co., Ltd (Hunan, China) and housed at the Department of Laboratory Animals, Central South University. These mice were maintained in sterile IVC cages under SPF conditions, with unrestricted access to sterilized food and water. For the establishment of the xenograft model, each mouse received a subcutaneous injection of 6 × 10^6^ AGS cells suspended in PBS containing 30% BD Matrigel. Tumour formation was monitored every week, with concurrent measurements of tumour volume and body weight. Tumour volume was computed as 0.5 × length × width^2^. Upon reaching a tumour volume of approximately 50 mm^3^, mice were arbitrarily assigned to two cohorts (n = 5 per group) (1): the ASO-NC group and (2) the ASO-circRERE(4–5) group. The ASO-circRERE(4-5) group received intratumoral injections of *in vivo*-optimized ASOs (5 nmol per administration, RiboBio) targeting circRERE(4-5) every 3 days, whereas the ASO-NC group received control ASOs (21, 22). After 14 days of treatment, mice were euthanized and tumours harvested for further analysis.

For metastasis models, 3 × 10^6^ AGS cells, which were labelled with firefly luciferase for *in vivo* tracking, were administered via tail vein injection to NOD-SCID mice. Following a one-week period, the mice underwent randomization into two groups (n = 5 per group) namely: an ASO-NC group and an ASO-circRERE(4-5) group. Mice in the ASO-circRERE(4-5) group were intravenously injected with circRERE(4-5)- targeting ASOs (10 nmol per injection, RiboBio) every 3 days, while the ASO-NC group received control ASOs (21, 23). Observations were conducted every week, with mouse weights recorded weekly. The dissemination of GC cells was tracked weekly using bioluminescence imaging utilizing the IVIS^®^ Spectrum imaging system (PerkinElmer). Bioluminescent signals were captured by administering 150 mg/kg of the luciferase substrate D-luciferin (YEASEN, 40902ES03) after intraperitoneal injection 10 minutes prior to imaging. At 5 weeks post-injection, mice were euthanized under humane protocols and lung tissues were harvested for further analysis. All procedures involving animals received ethical approval from the Department of Laboratory Animals, Central South University (Changsha, Hunan, China) (No. XMSB-2024-0220) and complied with the National Institutes of Health Guide for the Care and Use of Laboratory Animals.

### Statistical analysis

All non-animal experiments were independently conducted at least 3 times, with representative results presented. Data are expressed as the mean ± standard deviation (SD) based on a minimum of 3 biological replicates. Statistical differences were determined using either Student’s *t*-test or one-way or two-way analysis of variance (ANOVA), as indicated in the corresponding figure legends. Diagnostic performance of circRERE(4-5) as a GC biomarker was assessed using receiver operating characteristic (ROC) curve analysis, including calculation of area under the curve (AUC) values. All statistical analyses were performed using GraphPad Prism version 8.0. Significance thresholds were annotated in figures as follows: * *P <* 0.05; ** *P <* 0.01; *** *P <* 0.001; **** *P <* 0.0001.

## Results

### Upregulated circRERE(4-5) was associated with GC growth and metastasis

**Figure 1:**
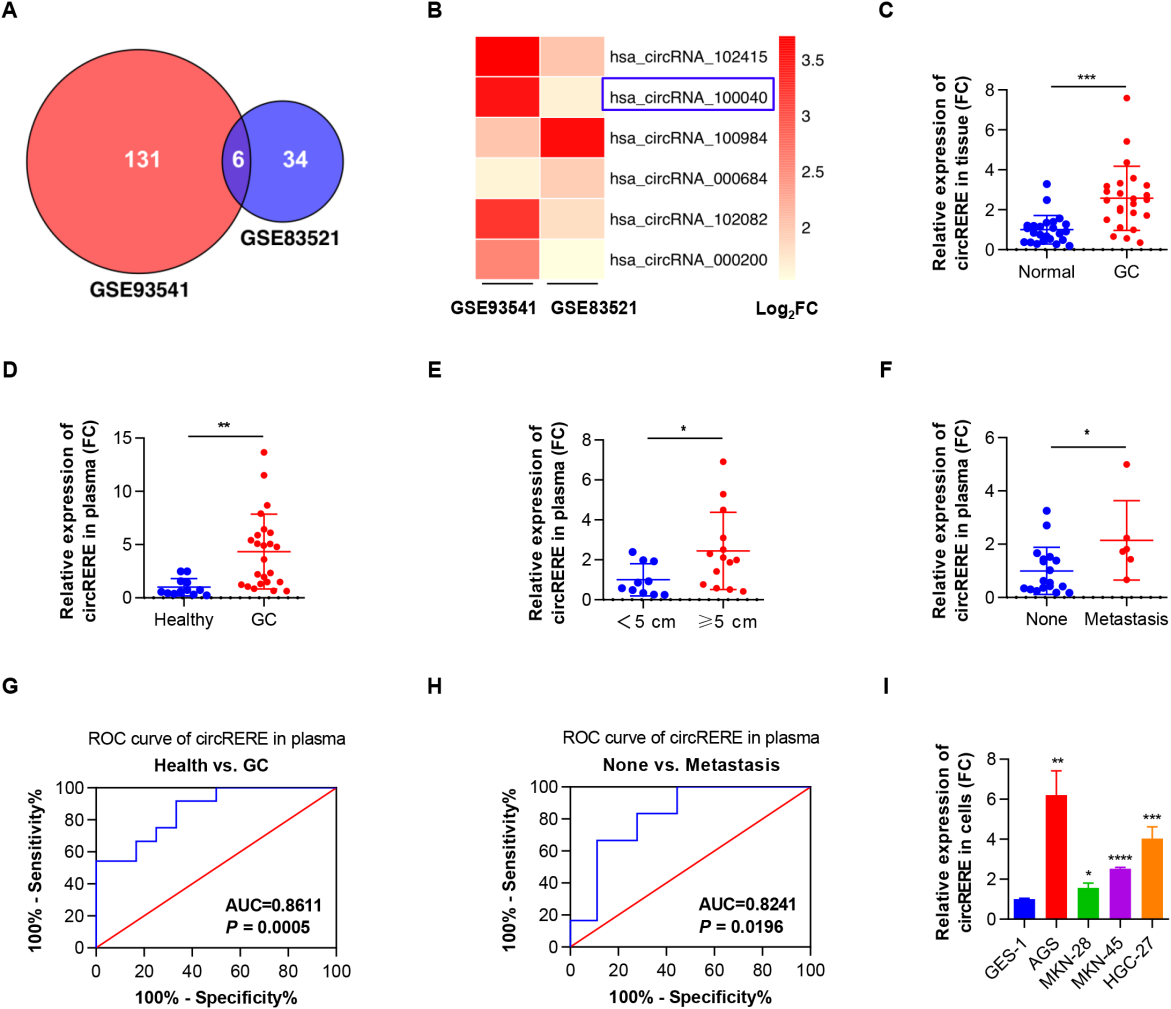
Upregulated circRERE(4-5) is associated with GC growth and metastasis. **(A)** A Venn diagram showing the overlap of differentially expressed circRNAs identified in the two microarray datasets. **(B)** A volcano plot highlighting 6 differentially expressed circRNAs across the two microarray datasets. **(C)** qRT–PCR assessment of circRERE(4-5) expression in 24 paired samples of GC tissues and adjacent normal tissues. **(D)** qRT–PCR assessment of circRERE(4-5) expression in plasma from 24 GC patients and 12 healthy controls. **(E)** qRT–PCR comparison of circRERE(4-5) expression in plasma from 10 GC patients with small tumours (< 5 cm) and 14 GC patients with large tumours (≥ 5 cm). **(F)** qRT–PCR comparison of circRERE(4-5) expression in plasma from 18 GC patients without distant metastases and 6 GC patients with distant metastases. **(G)** ROC curve analysis evaluating the diagnostic accuracy of circRERE(4-5) in plasma samples for differentiating GC patients from healthy controls. **(H)** ROC curve analysis evaluating the diagnostic accuracy of circRERE(4-5) in plasma samples for differentiating GC patients with distant metastases from those without. **(I)** qRT–PCR assessment of circRERE(4-5) expression in a gastric normal epithelial cell line (GES-1) and various GC cell lines. Data are shown as the mean ± SD. *P*-values were determined using a two-tailed paired **(C)** or unpaired Student’s *t*-test **(D-F)** or one-way ANOVA **(I)**; *P < 0.05, **P < 0.01, ***P < 0.001, ****P < 0.0001. See also **Supplementary Figure 1**.

To profile circRNA expression in GC tissues and patient plasma, data from two microarray datasets (GSE83521 and GSE93541) were analysed. The analysis identified 40 and 137 significantly upregulated circRNAs (log_2_FC ≥ 1.5, *P* < 0.05) in tissue and plasma samples, respectively (**Supplementary Figures 1A, B**, **Supplementary Tables 2**, **S3**). Intersection of the two datasets revealed 6 overlapping circRNAs (**Figures 1A, B**), with hsa_circRNA_100040 (circRERE(4-5)) the top upregulated circRNA in GC tissues (**Supplementary Figure 1C**). Moreover, hsa_circRNA_100040 was unequivocally shown to be overexpressed in GC tissues in an additional microarray dataset (GSE194384) (**Supplementary Figure 1D**). Quantitative RT–PCR analysis of 24 GC tissue pairs further demonstrated increased circRERE(4-5) expression in tumour samples (**Figure 1C**). Similarly, plasma from individuals with GC displayed markedly elevated circRERE(4-5) levels relative to that obtained from healthy volunteers (**Figure 1D**). Plasma circRERE(4-5) levels were also positively linked to tumour size and distant metastasis in patients with GC (**Figures 1E, F**). ROC analysis yielded an AUC value of 0.8611 (with a 95% confidence interval ranging from 0.7369 to 0.9853, and a *P*-value of 0.0005), indicating robust discriminative power for distinguishing GC patients from healthy controls (**Figure 1G**). Moreover, the AUC for differentiating patients with GC with distant metastasis from those without was 0.8241 (95% CI: 0.6503 to 0.9978, *P* = 0.0196) (**Figure 1H**), underscoring the capability of circRERE(4-5) as a plasma-based liquid biopsy marker for GC. Moreover, the expression of circRERE(4-5) was found to be higher in GC cell lines (AGS, MKN-28, MKN-45 and HGC-27), compared to the normal gastric epithelial cell line GES-1 (**Figure 1I**). To sum up, circRERE(4-5) was upregulated in GC and had a significant association with tumour progression.

### CircRERE(4-5) represents an exonic circRNA predominantly found in the cytoplasm

**Figure 2:**
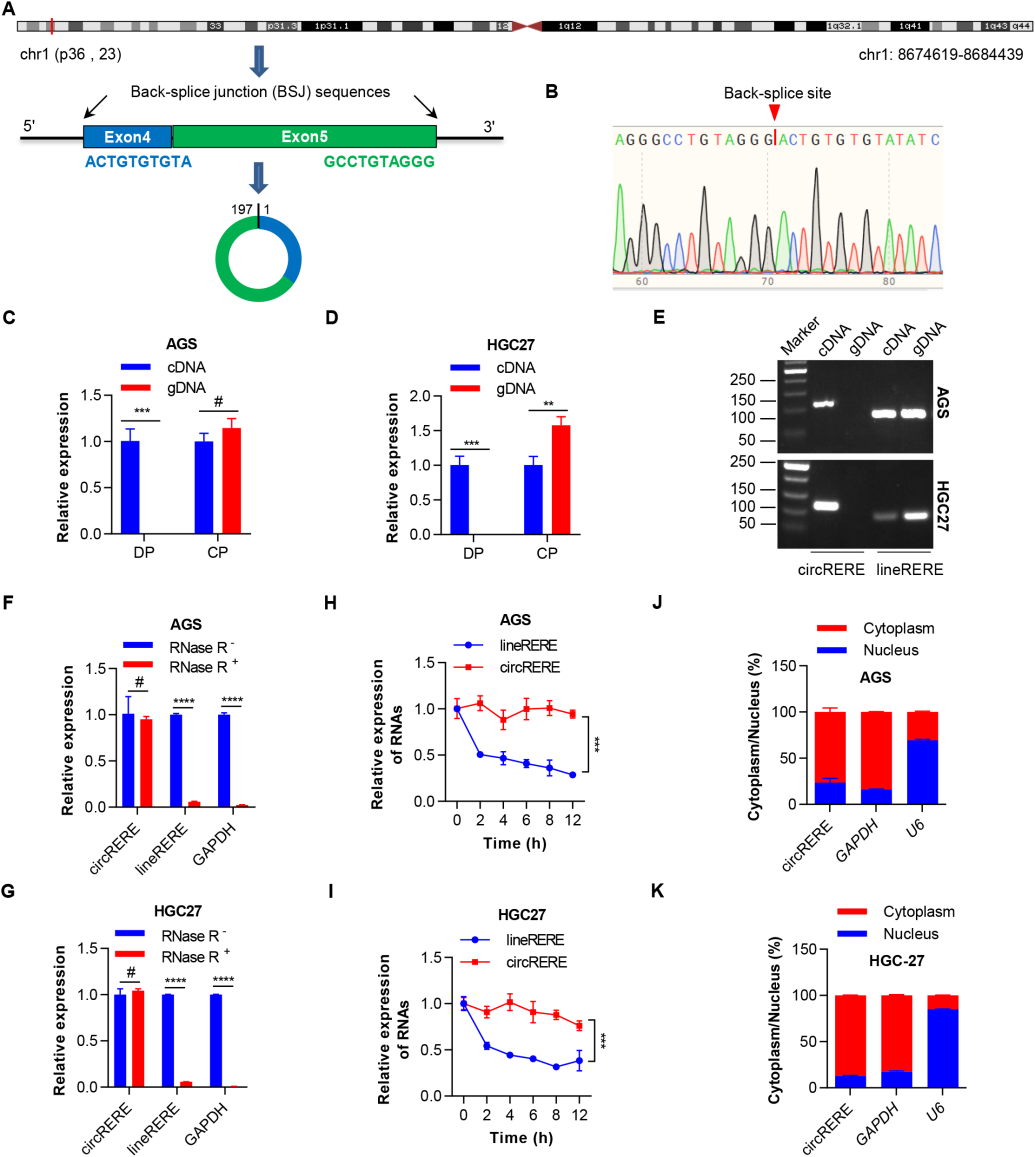
CircRERE(4-5) is a circRNA predominantly located in the cytoplasm. **(A)** Diagram illustrating the chromosomal location, exon structure and back-splice junction (BSJ) of circRERE(4-5). **(B)** Sanger sequencing results confirming the BSJ of circRERE(4-5). **(C, D)** qRT–PCR data demonstrating circRERE(4-5) amplification from complementary DNA (cDNA) and genomic DNA (gDNA) of GC cells using divergent primers (DP) and convergent primers (CP). **(E)** Agarose gel electrophoresis of PCR products confirming circRERE(4-5) circularization in GC cells. **(F, G)** qRT–PCR analysis of circRERE(4-5) and linear *RERE* (lineRERE) expression in GC cells after RNase R treatment (5 U/μg, 30 minutes). **(H, I)** qRT–PCR analysis of circRERE(4-5) and lineRERE expression in GC cells treated with actinomycin D (5 µg/mL) at different time points. **(J, K)** Cytoplasmic and nuclear RNA fractionation experiments indicating the localization of circRERE(4-5) in AGS and HGC-27 cells. *GAPDH* and U6 served as positive controls for the cytoplasm and nucleus, respectively. Data are shown as the mean ± SD. *P*-values were determined using a two-tailed unpaired Student’s *t*-test **(C, D, F, G)** or two-way ANOVA **(H, I)**; **P < 0.01, ***P < 0.001, ****P < 0.0001. See also **Supplementary Figure 2**.

According to circBase (circBase ID: hsa_circ_0009594) and circBank (circBank ID: hsa_RERE_0022200), circRERE(4-5) is an exon-exon junction circRNA situated on chromosome 1, specifically at the coordinates 8674619–8684439 and it consists of 197 nucleotides. Based on the human reference genome (GRCh37/hg19), circRERE(4-5) is derived from exons 4–5 within the arginine-glutamic acid dipeptide repeats (RERE) locus (**Figure 2A**), hence it is termed circRERE(4-5). Given that circRERE(4-5) has not been previously characterized, experiments were performed to confirm its existence and circularization. First, Sanger sequencing validated the BSJ site of circRERE(4-5) (**Figure 2B**), the sequence of which was consistent with that of circRERE(4-5) annotations in the circBank database and that of the circRERE(4-5) probe used in the circRNA microarray analysis. To exclude potential genomic rearrangements, divergent primers (DP) specific to circRERE(4-5), and convergent primers (CP) for linear *RERE* (lineRERE), were designed (**Supplementary Figures 2A, B**). The amplification of circRERE(4-5) occurred exclusively with DP in cDNA samples, while no amplification was observed in genomic DNA, validating the circular nature of *RERE* exons and eliminating the possibility of trans-splicing products (**Figures 2C–E**). The stability of circRERE(4-5) was confirmed through RNase R exonuclease treatment, which showed that circRERE(4-5) exhibited greater resistance to RNase R exonuclease degradation compared to its linear counterpart in AGS and HGC27 cells (**Figures 2F, G**). Furthermore, the use of the transcriptional inhibitor actinomycin D revealed that circRERE(4-5) had a longer half-life than lineRERE in these cell lines (**Figures 2H, I**). Subcellular localization by nucleocytoplasmic fractionation (**Figures 2J, K**) revealed predominant cytoplasmic enrichment of circRERE(4-5) in GC cells. To explore how circRERE(4-5) is exported from the nucleus, we examined the effects of exportin-2 (XPO2) and exportin-4 (XPO4)—two proteins known to mediate the nuclear export of circular RNAs (24, 25)—on the nucleo-cytoplasmic transport of circRERE(4-5). The results showed that siRNA-mediated knockdown of exportin-2 markedly decreased cytoplasmic circRERE(4-5) levels (**Supplementary Figures 2C–E**), whereas depletion of exportin-4 left cytoplasmic circRERE(4-5) abundance unchanged (**Supplementary Figures 2F–H**). These data indicate that circRERE(4-5) is exported by exportin-2. Collectively, these results identified circRERE(4-5) as a highly stable, cytoplasm-localized circRNA derived from the *RERE* gene in GC cells.

### Silencing of circRERE(4-5) inhibited the proliferation and migration of GC cells

**Figure 3:**
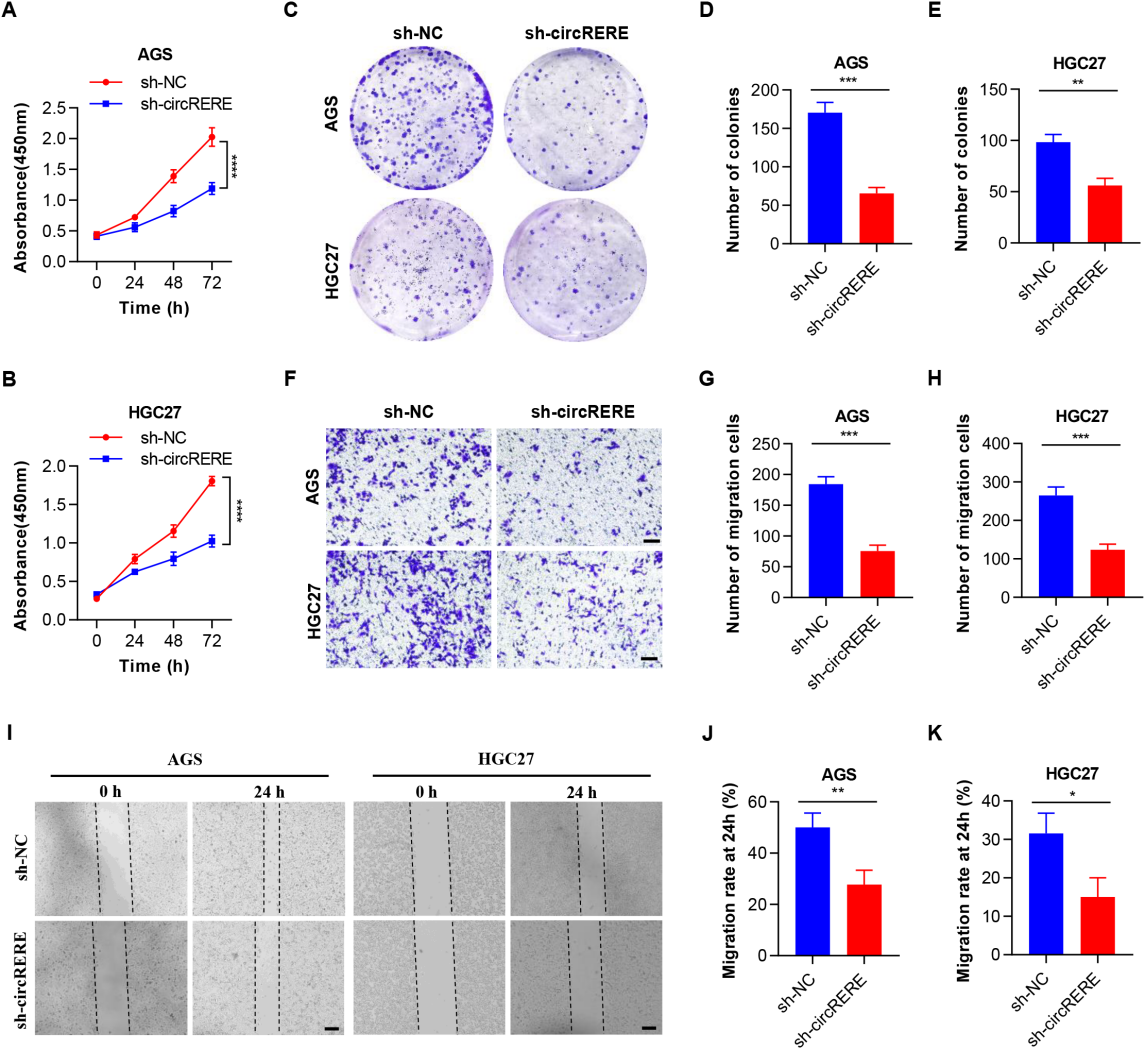
Knockdown of circRERE(4-5) suppresses GC cell proliferation and migration. **(A, B)** CCK-8 assay depicting the viability of AGS and HGC-27 cells under control (sh-NC) or circRERE(4-5) knockdown (sh-circRERE(4-5)) conditions at specified time points (0, 24, 48 and 72 hours). **(C-E)** Plate colony formation assay measuring colony formation in AGS and HGC-27 cells under control (sh-NC) or circRERE(4-5) knockdown (sh-circRERE(4-5)) conditions over 7–10 days. **(F-H)** Transwell migration assay assessing the migratory capacity of AGS and HGC-27 cells under control (sh-NC) or circRERE(4-5) knockdown (sh-circRERE(4-5)) conditions over 24 hours. Scale bar, 100 µm. **(I-K)** Wound healing assay evaluating the migratory capacity of AGS and HGC-27 cells under control (sh-NC) or circRERE(4-5) knockdown (sh-circRERE(4-5)) conditions over 24 hours. Scale bar, 200 µm. Data are shown as the mean ± SD. *P*-values were determined using a two-tailed unpaired Student’s *t*-test **(D, E, G-K)** or two-way ANOVA **(A, B)**; *P < 0.05, **P < 0.01, ***P < 0.001, ****P < 0.0001. See also **Supplementary Figure 3**.

To examine the involvement of circRERE(4-5) in GC progression, three shRNAs targeting the BSJ of circRERE(4-5) were designed to knock down effectively its expression in AGS and HGC27 cells (**Supplementary Figures 3A, B**). We then selected sh-circRERE-1, which exhibited the best knock-down efficiency, for subsequent functional and mechanistic studies. Notably, silencing circRERE(4-5) had minimal effects on the expression of *RERE* mRNA and other circRNAs derived from the *RERE* gene, including circRERE (3) (circBase ID: hsa_circ_0114356; circBank ID: hsa_RERE_0024600) (26), circRERE(4–10) (no circBase ID and circBank ID available at present) (27), circRERE(4–11) (circBase ID: hsa_circ_0009582; circBank ID: hsa_RERE_0009500) (28, 29), and circRERE (8–11) (circBase ID: hsa_circ_0009581; circBank ID: hsa_RERE_0009100) (30, 31) (**Supplementary Figures 3C, D**). Moreover, the WB results confirmed that RERE protein levels remain unchanged upon circRERE(4-5) knock-down (**Supplementary Figures 3E, F**), strengthening our conclusion that the observed phenotypes are specifically attributable to circRERE(4-5) rather than alterations in the host gene. As expected, CCK-8 (**Figures 3A, B**) and colony formation assays (**Figures 3C–E**) revealed a marked reduction in cell viability and proliferation following circRERE(4-5) knockdown. Additionally, the transwell migration assay (**Figures 3F–H**) and the wound healing assay (**Figures 3I–K**) indicated that depletion of circRERE(4-5) compromised the migratory ability of GC cells. Conversely, overexpression of circRERE(4-5) significantly enhanced the proliferation and migration of GC cells (**Supplementary Figures 3G–K**). Collectively, these results underscore the cancer-promoting function of circRERE(4-5) in GC.

### CircRERE(4-5) upregulated *ONECUT2* by sponging miR-571

**Figure 4:**
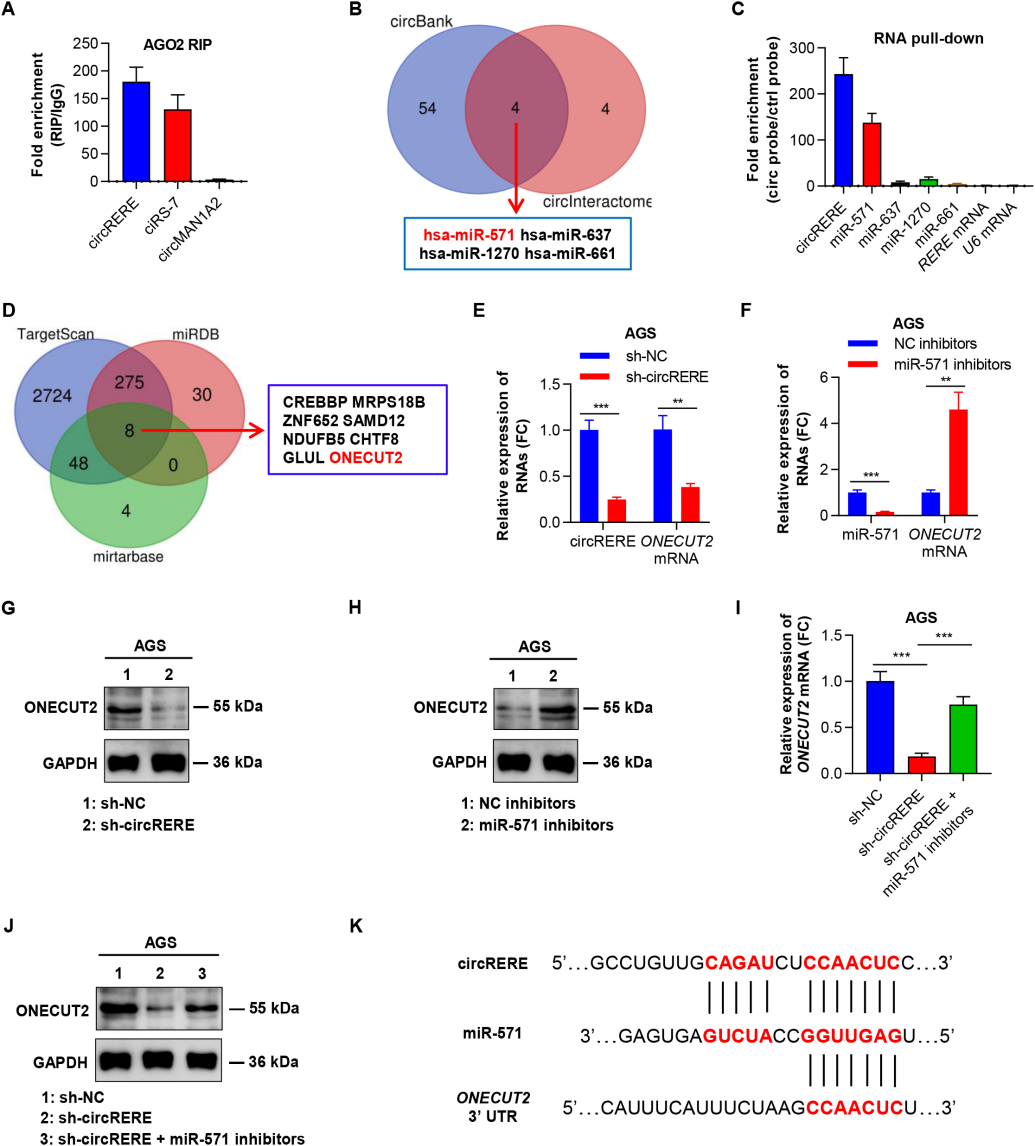
CircRERE(4-5) upregulates ONECUT2 by sponging miR-571. **(A)** qRT–PCR demonstrating the enrichment of circRNAs in a representative anti-AGO2 RIP assay conducted in GC cells. **(B)** Venn diagram illustrating the overlap of candidate miRNAs predicted to interact with circRERE(4-5) by two open-access databases. **(C)** qRT–PCR showing the enrichment of miRNAs following circRERE(4-5) pull-down in lysates of GC cells. **(D)** Venn diagram depicting the intersection of candidate mRNAs targeted by miR-571 as predicted by three open-access databases. **(E)** qRT–PCR analysis of *ONECUT2* mRNA expression in GC cells under control conditions (sh-NC) or with circRERE(4-5) knockdown (sh-circRERE(4-5)). **(F)** qRT–PCR analysis of *ONECUT2* mRNA expression in GC cells transfected with control inhibitors or miR-571 inhibitors. **(G)** Western blotting revealing ONECUT2 protein levels in GC cells under control conditions (sh-NC) or with circRERE(4-5) knockdown (sh-circRERE(4-5)), with GAPDH serving as a loading control. **(H)** Western blotting showing ONECUT2 protein levels in GC cells transfected with control inhibitors or miR-571 inhibitors. **(I)** qRT–PCR analysis of *ONECUT2* mRNA expression in AGS cells under control conditions (sh-NC), after circRERE(4-5) knockdown (sh-circRERE(4-5)), or following cotransfection with sh-circRERE(4-5) and miR-571 inhibitors. **(J)** Representative western blot of ONECUT2 in AGS cells under control conditions (sh-NC), after circRERE(4-5) knockdown (sh-circRERE(4-5)), or following cotransfection with sh-circRERE(4-5) and miR-571 inhibitors. **(K)** Sequence alignment analysis highlighting the binding sites of circRERE(4-5) and *ONECUT2* 3’ UTR on miR-571. Data are presented as the mean ± SD. *P*-values were calculated using a two-tailed unpaired Student’s *t*-test **(E, F, I)**; **P < 0.01, ***P < 0.001. See also **Supplementary Figure 4**.

To uncover how circRERE(4-5) influences cell proliferation and migration in GC, we examined its interactions with miRNAs, a typical way in which circRNAs affect gene expression (32–34). Initially, anti-AGO2 RIP assays were performed to determine if circRERE(4-5) acted as a miRNA sponge in GC cells. AGO2-IP efficiency was confirmed by western blot, which showed ≥ 12-fold enrichment of AGO2 relative to the IgG control (**Supplementary Figure 4A**). The findings indicated that circRERE(4-5) and ciRS-7 (a circRNA known to bind AGO2) (32, 35) were notably enriched by the anti-AGO2 antibody, whereas circMAN1A2 (which does not bind AGO2) (36) was not (**Figure 4A**). This finding implies that circRERE(4-5) likely interacts with miRNAs. Subsequently, the circBank and circInteractome databases were used to identify potential miRNAs that could bind to circRERE(4-5) (**Supplementary Tables 4**, **S5**). This search yielded 4 candidate miRNAs: hsa-miR-571; hsa-miR-637; hsa-miR-1270; and hsa-miR-661 (**Figure 4B**). qRT-PCR analysis revealed that miR-571 exhibited the highest expression abundance in gastric cancer cells compared with the other three miRNAs (**Supplementary Figure 4B**). To further confirm experimentally the miRNA that was interacting with circRERE(4-5) in GC cells, RNA pull-down assays, employing a biotin-labelled circRERE(4-5) probe, were performed. Endogenous circRERE(4-5) and miR-571, but not *RERE* mRNA and another 3 miRNAs, were notably enriched by the circRERE(4-5) probe relative to the control probe (**Figure 4C**). Moreover, subcellular fractionation assays showed that miR-571 was predominantly localized in the cytoplasm of GC cells, consistent with the subcellular distribution of circRERE(4-5) (**Supplementary Figure 4C**). These findings indicated that circRERE(4-5) could function as a molecular sponge for miR-571 in GC cells.

Subsequently, our objective was to pinpoint the target gene of miR-571 in GC cells. Drawing on the TargetScan, miRDB, and miRTarBase databases, we identified 8 genes – namely *ONECUT2*, *CREBBP*, *MRPS18B*, *ZNF652*, *SAMD12*, *NDUFB5*, *CHTF8*, and *GLUL* – as potential targets of miR-571 (**Figure 4D**, **Supplementary Tables 6**-**S8**). Among these genes, only *ONECUT2* mRNA was significantly downregulated after circRERE(4-5) knockdown (**Figure 4E**, **Supplementary Figure S4D**) and upregulated with miR-571 inhibition in GC cells (**Figure 4F**, **Supplementary Figure S4E**), as shown by qRT–PCR. Western blot analysis further validated that silencing circRERE(4-5) led to a decrease in ONECUT2 protein levels (**Figure 4G**). Conversely, inhibiting miR-571 resulted in elevated ONECUT2 protein expression in GC cells (**Figure 4H**). Of note, the reduction in *ONECUT2* mRNA and protein expression caused by circRERE(4-5) knockdown was reversed by miR-571 inhibitors (**Figures 4I, J**, **Supplementary Figures S4F, G**). Moreover, sequence alignment analysis demonstrated that both circRERE(4-5) and the 3’ UTR of *ONECUT2* mRNA possess identical binding sites for miR-571 (**Figure 4K**). Collectively, these findings suggest that circRERE(4-5) may function as a molecular sponge for miR-571, thereby upregulating ONECUT2 in GC.

### CircRERE(4-5) exerted its oncogenic effect via ONECUT2 in GC cells

**Figure 5:**
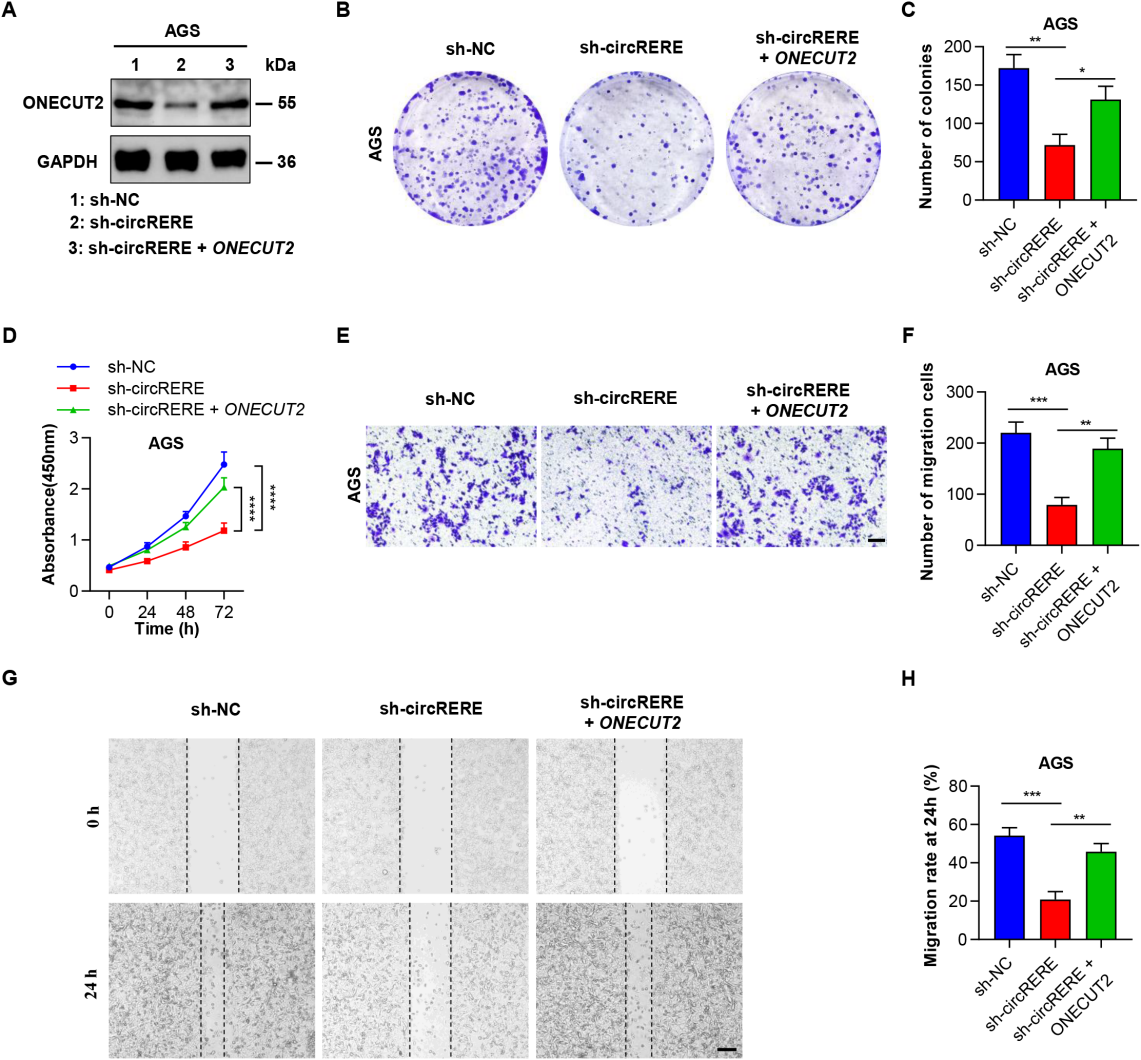
CircRERE(4-5) promotes oncogenic activity through ONECUT2 in GC cells. **(A)** Representative western blot of ONECUT2 protein in AGS cells under control conditions (sh-NC), after circRERE(4-5) knockdown (sh-circRERE(4-5)), or following cotransfection with sh-circRERE(4-5) and *ONECUT2* vector. **(B, C)** Plate colony formation assay evaluating colony formation in AGS cells under control conditions (sh-NC), after circRERE(4-5) knockdown (sh-circRERE(4-5)), or following cotransfection with sh-circRERE(4-5) and *ONECUT2* vector. **(D)** CCK-8 assay depicting the proliferation of AGS cells under control conditions (sh-NC), after circRERE(4-5) knockdown (sh-circRERE(4-5)), or following cotransfection with sh-circRERE(4-5) and *ONECUT2* vector. **(E, F)** Transwell migration assay illustrating the migration of AGS cells under control conditions (sh-NC), after circRERE(4-5) knockdown (sh-circRERE(4-5), or following cotransfection with sh-circRERE(4-5) and *ONECUT2* vector. Scale bar, 100 µm. **(G, H)** Wound healing assay demonstrating the migration of AGS cells under control conditions (sh-NC), after circRERE(4-5) knockdown (sh-circRERE(4-5), or following cotransfection with sh-circRERE(4-5) and ONECUT2 vector. Scale bar, 200 µm. Data are presented as the mean ± SD. *P-*values were calculated using a two-tailed unpaired Student’s *t*-test **(C, F, H)** or two-way ANOVA **(D)**; *P < 0.05, **P < 0.01, ***P < 0.001, ****P < 0.0001. See also **Supplementary Figure 5**.

Recent studies have demonstrated that ONECUT2 is upregulated during the tumorigenic process of GC, where it promotes cell proliferation, metastasis, stemness and oxaliplatin resistance in GC (37–39). To elucidate the role of ONECUT2 in the circRERE(4-5)-mediated oncogenic effect, *in vitro* rescue experiments were conducted. As shown in **Figure 5A**, **Supplementary Figure S5A**, ONECUT2 overexpression restored *ONECUT2* expression levels, which were reduced by circRERE(4-5) knockdown. Consistent with these observations, assays including the plate colony formation assay (**Figures 5B, C**, **Supplementary Figures S5B, C**), CCK-8 assay (**Figure 5D**), Transwell migration assay (**Figures 5E, F**, **Supplementary Figures S5D, E**) and the wound healing assay (**Figures 5G, H**) collectively indicated that overexpression of ONECUT2 mitigated the inhibitory effects of circRERE(4-5) knockdown on the proliferation and migration of GC cells. These data indicate that circRERE(4-5) facilitates GC progression by increasing the expression of ONECUT2.

### CircRERE(4-5) knockdown inhibited the tumorigenesis and metastasis of GC

**Figure 6:**
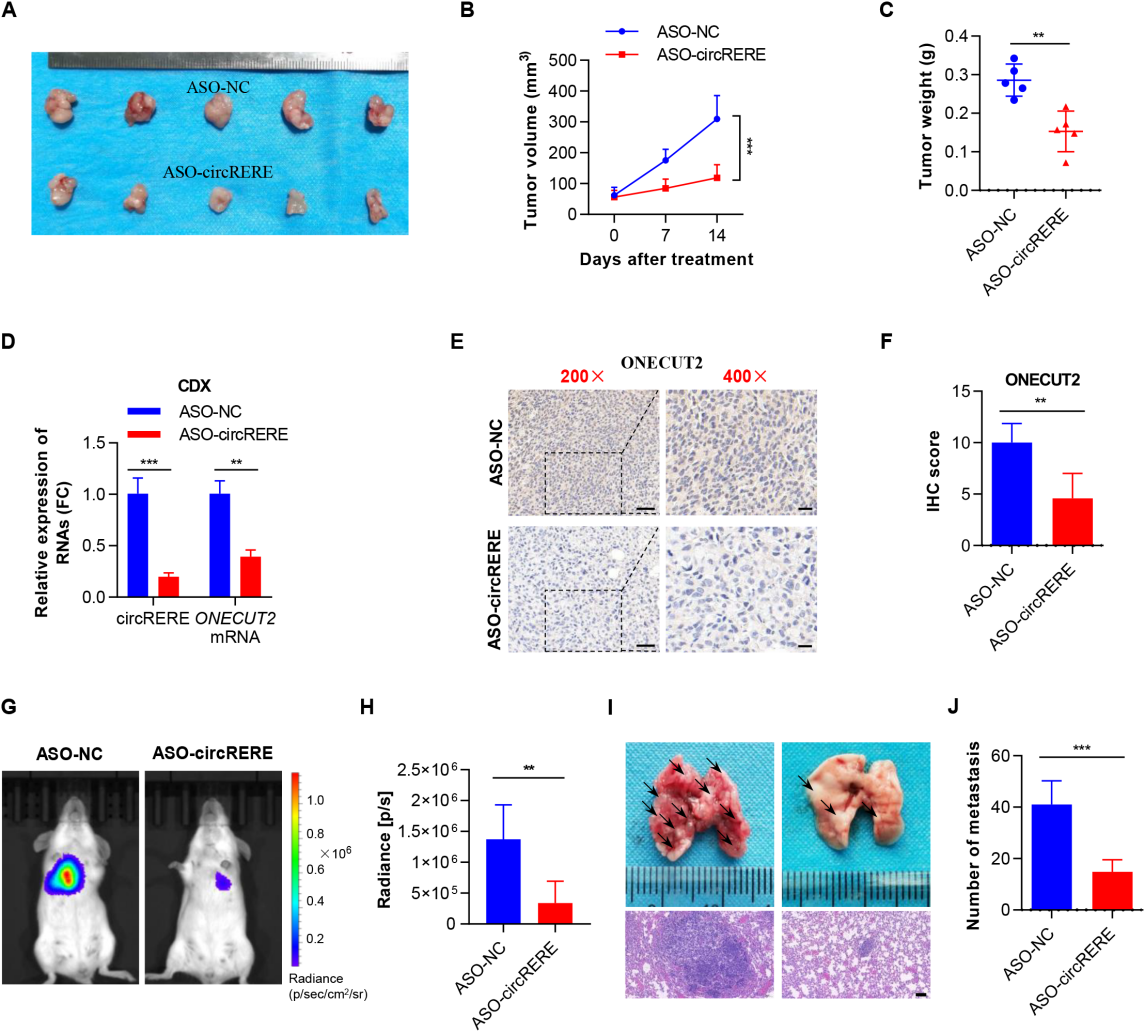
CircRERE(4-5) knockdown suppresses GC tumorigenesis and metastasis. **(A)** Tumour images of AGS cell derived xenograft **(CDX)** from mice receiving intratumoral injections of control ASOs or *in vivo*-optimized circRERE(4-5) ASOs. **(B)** Tumour growth trajectories in each group of mice. **(C)** Tumour weights in each group of mice. **(D)** qRT–PCR demonstrating the expression levels of circRERE(4-5) and *ONECUT2* mRNA in tumours from control (ASO-NC) or circRERE(4-5) knockdown (ASO-circRERE(4-5)) groups. **(E, F)** IHC staining illustrating the expression levels of ONECUT2 protein in tumours from control (ASO-NC) or circRERE(4-5) knockdown (ASO-circRERE(4-5)) groups. Scale bar, 50 µm (left), 20 µm (right). **(G, H)** The dissemination of AGS cells in control (ASO-NC) or circRERE(4-5) knockdown (ASO-circRERE(4-5)) groups was visualized by bioluminescence imaging using the IVIS^®^ Spectrum imaging system. Bioluminescent signals were captured after intraperitoneal injection of 150 mg/kg D-luciferin 10 minutes prior to imaging. **(I)** Lung metastasis nodules in mice receiving intravenous injections of control ASOs or *in vivo*-optimized circRERE(4-5) ASOs. Scale bar, 100 µm. **(J)** The count of lung metastasis nodules in control (ASO-NC) or circRERE(4-5) knockdown (ASO-circRERE(4-5)) groups. Data are presented the as mean ± SD. *P*-values were calculated using a two-tailed unpaired Student’s *t*-test **(C, D, F, H, J)** or two-way ANOVA **(B)**; **P < 0.01, ***P < 0.001. See also **Supplementary Figure 6**.

To evaluate the impact of circRERE(4-5) on GC tumorigenesis and metastasis *in vivo*, subcutaneous xenograft and systemic metastasis mouse models were established, which were subsequently treated with *in vivo*-optimized ASOs targeting circRERE(4-5) (ASO-circRERE). First, we validated the knockdown efficiency of ASO-circRERE in GC cells *in vitro*. As shown in **Supplementary Figures 6A**, the ASO-circRERE used for animal experiments achieved approximately 82% knockdown of circRERE(4-5) in AGS cells without altering linear *RERE* mRNA levels, thereby ruling out major off-target effects. Expectedly, xenograft tumours treated with circRERE(4-5)-targeted ASOs were significantly smaller and lighter than those treated with control ASOs (**Figures 6A–C**), indicating that circRERE(4-5) knockdown substantially inhibits GC cell growth *in vivo*. qRT–PCR analysis confirmed a marked reduction in circRERE(4-5) and *ONECUT2* mRNA levels in tumours from the ASO-circRERE(4-5) group compared to the ASO-NC group (**Figure 6D**). Consistently, immunohistochemical (IHC) staining showed decreased ONECUT2 protein expression in tumours following treatment of circRERE(4-5)-targeted ASOs (**Figures 6E, F**). For systemic metastasis models, intravenous administration of circRERE(4-5)-targeted ASOs demonstrated effective suppression of GC cell spread (**Figures 6G, H**), reducing metastatic tumour nodules in the lungs compared to control ASOs (**Figures 6I, J**). These results strongly suggest that circRERE(4-5)-targeted therapy can inhibit GC growth and metastasis *in vivo* by suppression of ONECUT2.

## Discussion

Recent studies have linked circRNAs to the biological regulation of cancer metastasis (40–43). However, the molecular mechanisms by which circRNAs modulate GC progression remain incompletely understood, and no circRNA-based therapeutics have yet gained clinical approval (44, 45). The present study identifies circRERE (4–5) as a metastasis-associated circRNA that significantly promotes GC growth and metastasis through a competing endogenous RNA (ceRNA) mechanism involving miR-571 sequestration and consequent ONECUT2 upregulation. Importantly, our animal model findings support circRERE(4-5) as a promising target for preventing metastatic spread in GC.

According to the circBank database, the human *RERE* gene yields approximately 314 distinct circRNAs through alternative back-splicing. Prior studies have characterized circRERE(4-10) in colorectal cancer ferroptosis regulation (27), circRERE(3) in antitumor immunity (26), and circRERE (4–11) in hepatocellular carcinoma proliferation and invasion (28). Our work introduces circRERE(4-5) as a functionally independent entity in GC, with distinct molecular partners (miR-571/ONECUT2) and biological outputs (metastatic colonization) that do not overlap with previously described *RERE* circRNAs. Actually, circRERE(4-5) was previously identified as a circulating biomarker in GC (46); here we provide functional and mechanistic extension of that observation. This “same gene, different circRNAs, distinct functions” pattern underscores the importance of precise circRNA isoform annotation in functional studies, rather than collapsing all *RERE*-derived transcripts into a single regulatory category.

Our bioinformatics analyses predicted multiple miRNAs capable of binding circRERE(4-5), yet RNA pull-down experiments identified miR-571 as the predominant interacting partner. Several factors may explain this selectivity. First, the circRERE(4-5) back-splice junction creates a unique structural context that positions the miR-571 binding site in an accessible, single-stranded loop region, whereas other predicted sites may be sequestered in double-stranded stems. Second, miR-571 exhibits relatively high abundance in GC cells compared with other candidates (miR-637, miR-1270, miR-661), satisfying the “ceRNA stoichiometry” requirement that sponge efficacy depends on target availability. Third, miR-571 preferentially localizes to cytoplasmic processing bodies, overlapping with the predominantly cytoplasmic distribution of circRERE(4-5) that we observed by subcellular fractionation. This spatial co-localization likely facilitates stable RNA-RNA interaction. Notably, miR-571 has been independently reported to suppress metastasis or prevent aberrant DNA replication in multiple cancer types by targeting oncogenes (47, 48), consistent with our observation that miR-571 overexpression phenocopies circRERE(4-5) knockdown. Thus, the circRERE(4-5)/miR-571 pairing represents a functionally relevant, stoichiometrically feasible, and spatially compatible regulatory axis rather than a bioinformatic artifact.

ONECUT2 (also known as OC2) is a transcription factor belonging to the human one-cut domain family that governs diverse biological processes by modulating downstream targets in various malignancies such as prostate (49–52), breast (53), colorectal (54), hepatocellular (55), and ovarian cancers (56). In gastric cancer (GC), ONECUT2 is frequently overexpressed and promotes tumour cell proliferation, stemness, chemoresistance, and immune cell infiltration within the tumour microenvironment (TME) (37, 38). The mechanisms underlying its dysregulated expression have been investigated at multiple levels. Seo et al. demonstrated that promoter-proximal CpG hypomethylation correlates with ONECUT2 upregulation in primary GC (39), while Lin et al. established that *Helicobacter pylori* (HP) infection activates the NF-κB pathway to enhance *ONECUT2* transcription (38). Post-transcriptionally, Fan and colleagues identified that YTHDF2, an m^6^A “reader” downregulated in GC, facilitates degradation of *ONECUT2* mRNA through m^6^A modification (37). The present study uncovers an additional layer of regulation via a ceRNA mechanism; specifically, circRERE(4–5) sequesters miR-571, thereby derepressing ONECUT2 expression. This finding expands the established ceRNA network, which already includes miR-15a-5p (57) and miR-202-3p (58)—the latter itself regulated by lncRNA DNAH17-AS1 (58). Collectively, the circRERE(4–5)/miR-571/ONECUT2 axis represents a critical node in the post-transcriptional regulatory circuitry driving GC tumorigenesis and metastasis.

Another important finding of our study is that preclinical efficacy data using *in vivo*-optimized ASOs targeting circRERE(4-5) support the translational potential of this therapeutic approach. ASO-mediated circRNA depletion achieved 80% knockdown in tumour tissue, comparable to genetic shRNA efficacy, with no detectable hepatotoxicity or renal toxicity at the doses tested. Over the past few decades, ASO-based drugs have rapidly evolved, demonstrating robust inhibitory potency, efficient cellular uptake, low toxicity and extended half-lives (59–61). Notably, several ASO drugs, such as vitravene and nusinersen, have received FDA approval for clinical use (62–64). Given their antitumor effects in animal models, ASOs hold promise as therapeutic agents for the treatment of cancer (65–70). Therefore, our findings suggest that circRERE(4-5)-targeted ASOs may serve as a novel therapeutic strategy for the treatment of GC.

The present research has certain limitations that warrant acknowledgment. Initially, although circRERE(4-5) is overexpressed in GC, we have not yet delved into the mechanisms driving its upregulation. Subsequently, while circRERE(4-5) shows potential as a biomarker for early GC diagnosis and prognostic assessment, its diagnostic and predictive capabilities require further validation using extensive clinical samples. The aforementioned limitations will guide our future research endeavours. Concurrently, we aspire to elucidate further the biological characteristics of circRERE(4-5) and to develop novel circRNA-based therapeutic agents.

## Conclusions

In summary, the present study has identified circRERE(4-5) as a potent metastasis-promoting circRNA in GC, underscoring its significant contribution to GC progression. Additionally, it was unequivocally demonstrated that circRERE(4-5) regulates the expression of *ONECUT2*, which is a target gene of miR-571. These insights potentially could guide diagnostic and therapeutic strategies for GC involving circRERE(4-5) silencing.

## Data Availability

The original contributions presented in the study are included in the article/**Supplementary Material**. Further inquiries can be directed to the corresponding authors.
