# Supplementary material for: Silencing of circRERE(4-5) inhibits ONECUT2-mediated tumorigenesis and metastasis in gastric cancer
Source: Front Immunol. 2026 Mar 6;17:1686702. doi: 10.3389/fimmu.2026.1686702 (PMC13002853; doi:10.3389/fimmu.2026.1686702)
Supplement: Supplementary file 1 [file DataSheet1.pdf]

# Supplementary Figures and Figure legends

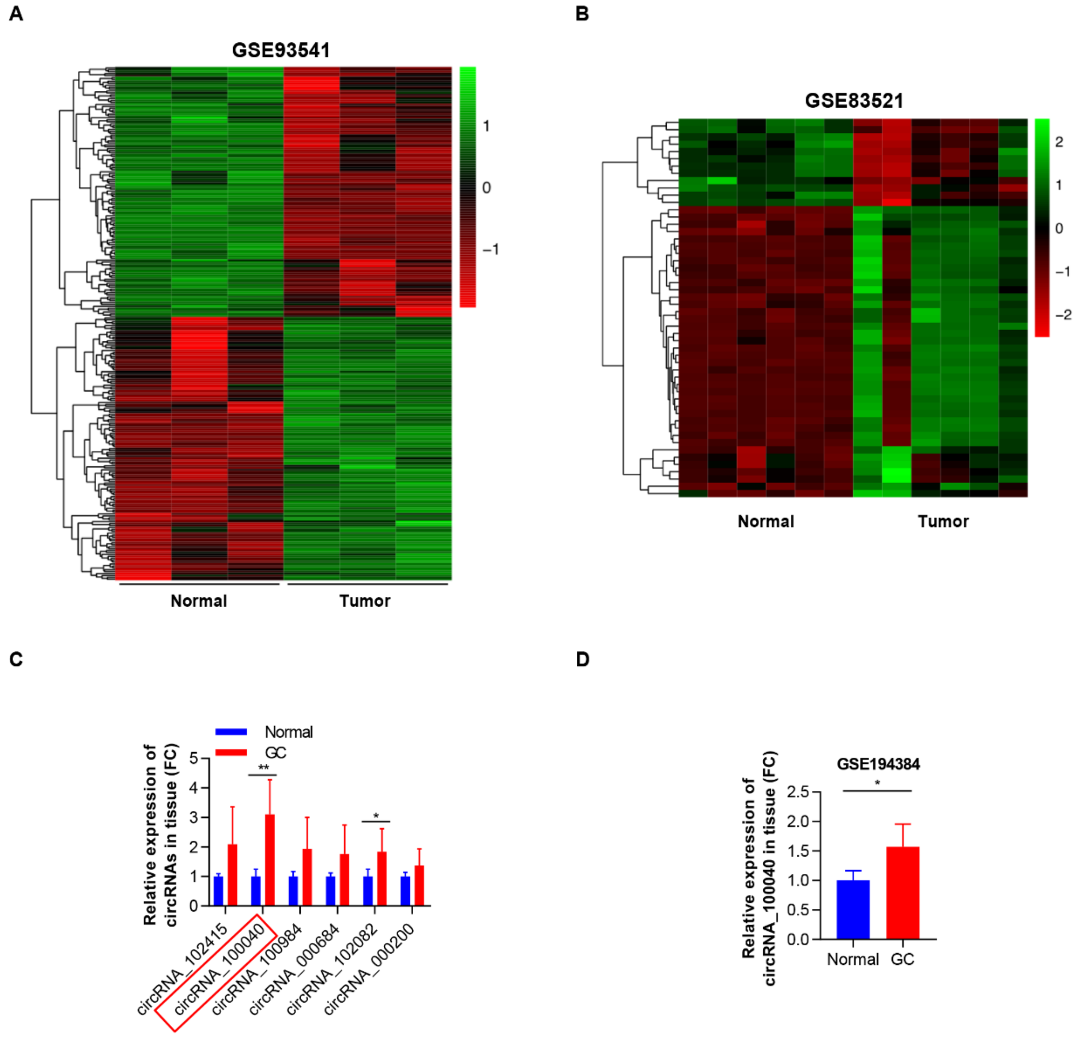


**Figure S1. Expression profile of circRERE(4-5) in GC.**

(**A**) Heatmap depicting differentially expressed circRNAs in plasma samples from 3 GC patients and 3 healthy controls (GSE93541). (**B**) Heatmap illustrating differentially expressed circRNAs in 6 paired GC and adjacent normal tissues (GSE83521). (**C**) qRT–PCR analysis of circRNA expression in 10 paired GC and adjacent normal tissues. (**D**) Relative expression levels of circRNA_100040 (circRERE(4-5)) in the GSE194384 dataset.

Notes: Data are presented as the mean ± SD. *P*-values were calculated using a two-tailed paired Student's *t*-test (C and D); *$P < 0.05$, **$P < 0.01$. Related to **Figure 1**.

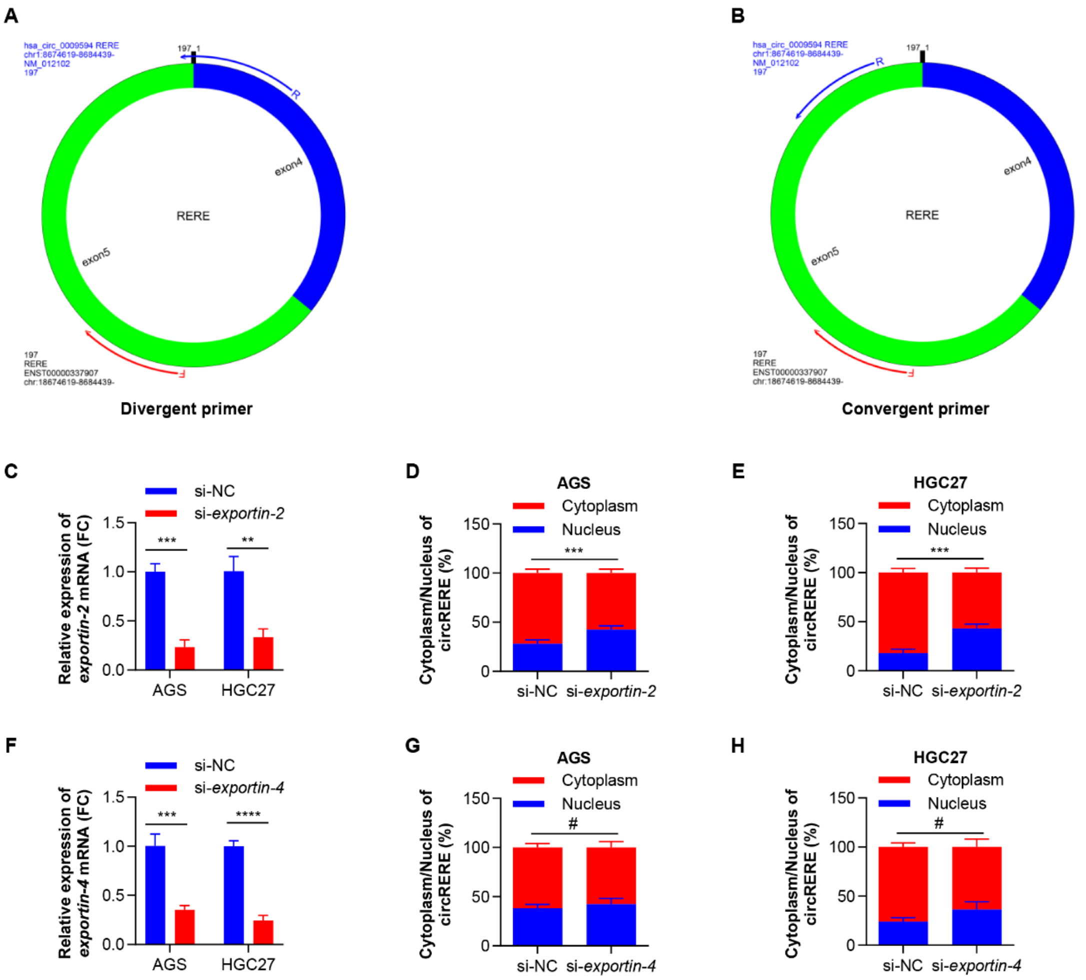


**Figure S2. Primer design and nuclear export of circRERE(4-5).**

(**A**) Schematic of divergent primer (DP) design for circRERE(4-5) using circPrimer software 2.0. (**B**) Schematic of convergent primer (CP) design for circRERE(4-5) using circPrimer software 2.0. (**C**) qRT–PCR analysis of the relative expression levels of *exportin-2* mRNA in GC cells under control conditions (si-NC) or with *exportin-2* knockdown (si-*exportin-2*). (**D, E**) Cytoplasmic and nuclear RNA fractionation experiments indicating the localization of circRERE(4-5) in GC cells under control conditions (si-NC) or with *exportin-2* knockdown (si-*exportin-2*). (**F**) qRT–PCR analysis of the relative expression levels of *exportin-4* mRNA in GC cells under control conditions (si-NC) or with *exportin-4* knockdown (si-*exportin-4*). (**G, H**) Cytoplasmic and nuclear RNA fractionation experiments indicating the localization of circRERE(4-5) in GC cells under control conditions (si-NC) or with *exportin-4* knockdown (si-*exportin-4*).

Notes: Data are shown as the mean ± SD. *P*-values were determined using a two-tailed unpaired Student's *t*-test (C-H); $**P < 0.01$, $***P < 0.001$, $****P < 0.0001$. Related to **Figure 2**.

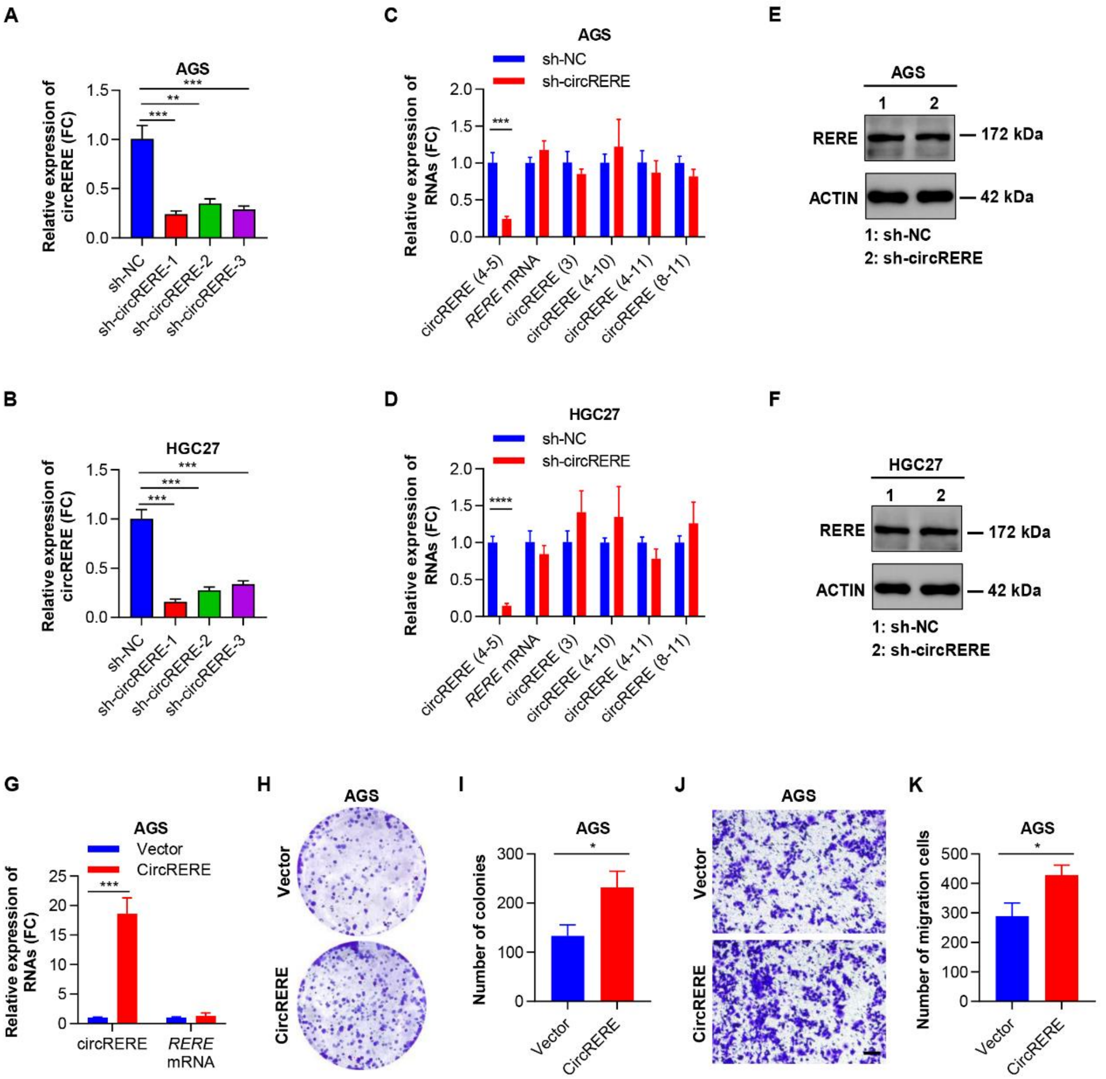


**Figure S3. RNA expression in GC cells following circRERE(4-5) knockdown (sh-circRERE)**

(**A, B**) qRT–PCR analysis of the expression levels of circRERE(4-5) in AGS and HGC27 cells under control conditions (sh-NC) or with circRERE(4-5) knockdown (sh-circRERE). (**C, D**) qRT–PCR analysis of the expression levels of circRERE(4-5), *RERE* mRNA and other RERE-derived circRNAs in AGS and HGC27 cells under control conditions or with circRERE(4-5) knockdown. (**E, F**) WB analysis of the expression levels of RERE protein in AGS and HGC27 cells under control conditions or with circRERE(4-5) knockdown. (**G**) qRT–PCR analysis of the relative expression levels of circRERE(4-5) in AGS cells under control conditions (Vector) or with circRERE(4-5) overexpression (CircRERE). (**H, I**) Plate colony formation assay measuring colony formation in AGS cells under control or circRERE(4-5) overexpression conditions over 7-10 days. (**J, K**) Transwell migration assay assessing the migratory capacity of AGS cells under control or circRERE(4-5) overexpression conditions over 24 hours. Scale bar, 100 μm.

Notes: Data are shown as the mean ± SD. *P*-values were determined using a two-tailed unpaired Student's *t*-test. *$P < 0.05$, ***$P < 0.001$, ****$P < 0.0001$. Related to **Figure 3**.

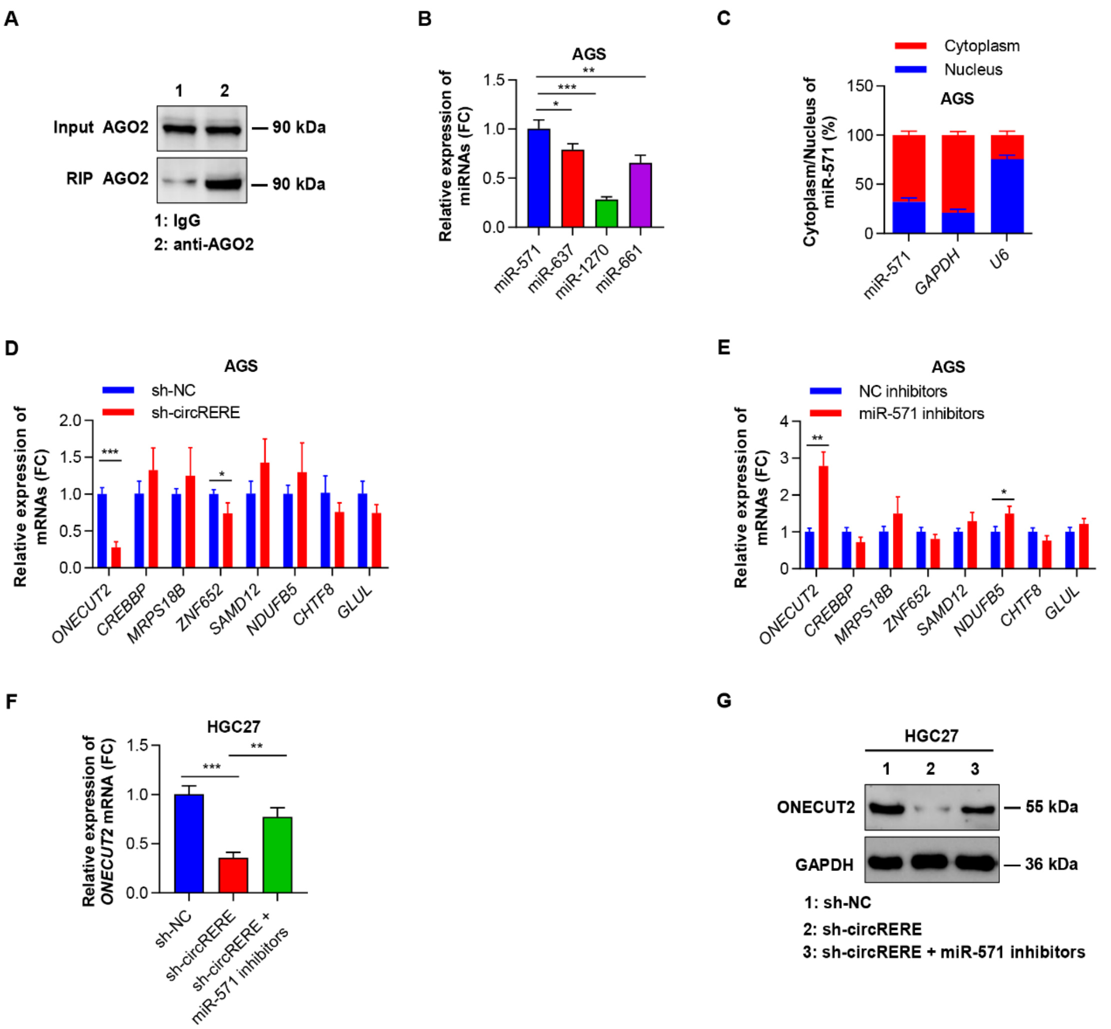


**Figure S4. ONECUT2 expression in GC cells with circRERE(4-5) knockdown or miR-571 inhibition**

(**A**) Western blotting showing AGO2 protein levels in GC cells following IgG-RIP or anti-AGO2-RIP. (**B**) qRT–PCR analysis of the expression levels of 4 miRNAs in AGS cells. (**C**) Cytoplasmic and nuclear RNA fractionation experiments indicating the localization of miR-571 in AGS cells. *GAPDH* and U6 served as positive controls for the cytoplasm and nucleus, respectively. (**D**) qRT–PCR analysis of selected gene expression in GC cells under control conditions (sh-NC) or with circRERE(4-5) knockdown (sh-circRERE). (**E**) qRT–PCR analysis of selected gene expression in GC cells after transfection with control inhibitors or miR-571 inhibitors. (**F**) qRT–PCR analysis of *ONECUT2* mRNA expression in HGC27 cells under control conditions (sh-NC), after circRERE(4-5) knockdown (sh-circRERE(4-5)), or following cotransfection with sh-circRERE(4-5) and miR-571 inhibitors. (**G**) Representative western blot of ONECUT2 in HGC27 cells under control conditions (sh-NC), after circRERE(4-5) knockdown (sh-circRERE(4-5)), or following cotransfection with sh-circRERE(4-5) and miR-571 inhibitors.

Notes: Data are presented as the mean ± SD. *P*-values were calculated using a two-tailed unpaired Student's *t*-test (B-F); **P* < 0.05, ***P* < 0.01, ****P* < 0.001. Related to **Figure 4**.

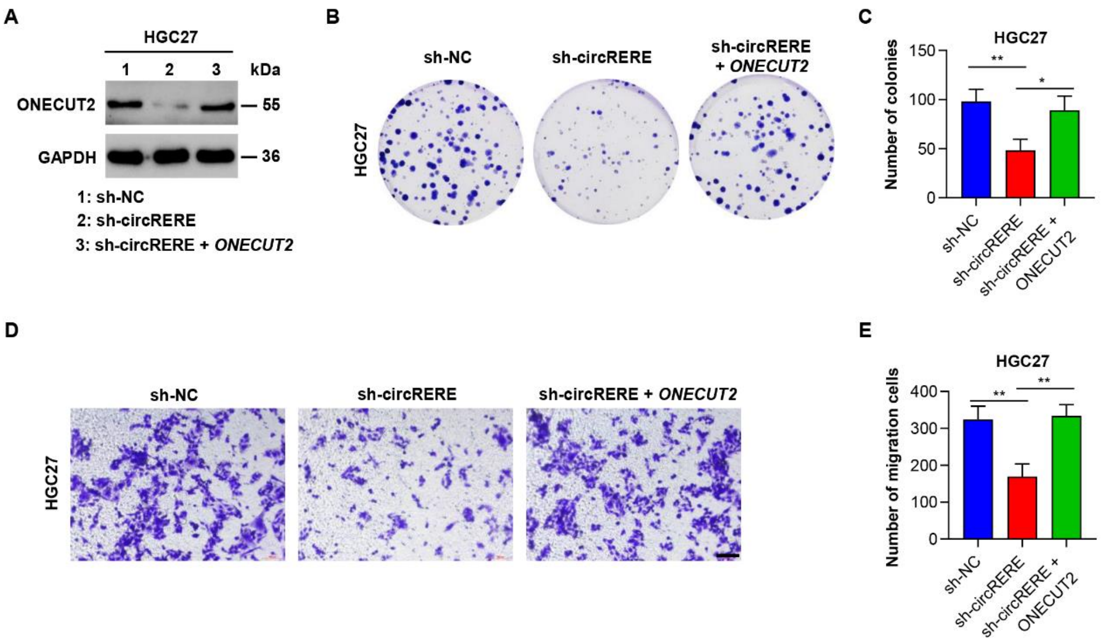


**Figure S5. CircRERE(4-5) exerts its oncogenic effect via ONECUT2**

(**A**) Representative western blot of ONECUT2 protein in HGC27 cells under control conditions (sh-NC), after circRERE(4-5) knockdown (sh-circRERE(4-5)), or following cotransfection with sh-circRERE(4-5) and *ONECUT2* vector. (**B, C**) Plate colony formation assay evaluating colony formation in HGC27 cells under control conditions (sh-NC), after circRERE(4-5) knockdown (sh-circRERE(4-5)), or following cotransfection with sh-circRERE(4-5) and *ONECUT2* vector. (**D, E**) Transwell migration assay illustrating the migration of HGC27 cells under control conditions (sh-NC), after circRERE(4-5) knockdown (sh-circRERE(4-5), or following cotransfection with sh-circRERE(4-5) and *ONECUT2* vector. Scale bar, 100 µm.

Notes: Data are presented as the mean ± SD. *P*-values were calculated using a two-tailed unpaired Student's *t*-test (C, E); *$P < 0.05$, **$P < 0.01$. Related to **Figure 5**.

A

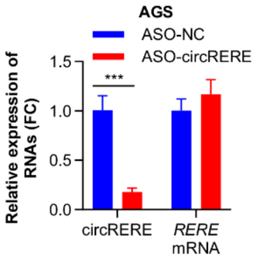


**Figure S6. RNA expression in GC cells following circRERE(4-5) knockdown (ASO-circRERE)**
(**A**) qRT–PCR analysis of the expression levels of circRERE(4-5) and *RERE* mRNA in AGS cells under control conditions (ASO-NC) or with circRERE(4-5) knockdown (ASO-circRERE).
Notes: Data are presented as the mean ± SD. *P*-values were calculated using a two-tailed unpaired Student's *t*-test (C, E); ***$P < 0.001$. Related to **Figure 6**.

# Supplementary Tables

**Table S1 Primers used in this article**

| Gene names | Forward primers (5'→3') | Reverse primers (5'→3') |
|---|---|---|
| circRERE(4-5) divergent primer | CCAACTCCTGCTTTGTGTGA | ACTCTCGATATACACACAGTCCC |
| circRERE(4-5) convergent primer | CCAACTCCTGCTTTGTGTGA | CCTCTCCCGGCTTCAGAAA |
| *RERE* mRNA | GCCCAGGCTTACTGTCTACC | CTCCTTCCAAGGTACAGCTCC |
| circRERE(3) | AGGACAGATACCGGTGAGAT | GGCCTCCGTGAAAGGTAGAC |
| circRERE(4-10) | AGTTGTGGACCTGTGTTCAG | TGCAGGAATGTGTGATGGAG |
| circRERE(4-11) | GAGTGGTGTTATCCCGAGAG | GGGAATTGGTTCATCATGCG |
| circRERE(8-11) | TTTGCAGGAATGTGTGATGG | ATGAATCCACTCGGGCTTTA |
| *ONECUT2* mRNA | CAAACGCCCGTCAAAGGAGAT | GCTCAGATCGTCTTGCCACTT |
| *GAPDH* | GGTCGGAGTCAACGGATTTG | ATGAGCCCCAGCCTTCTCCAT |
| *U6* | CGCTTCGGCAGCACATATAC | TTCACGAATTTGCGTGTCATC |
| miR-571 | ACACTCCAGCTGGGAGAGTTGGCCAT CTG | TGGTGGTCGTGGAGTCG |

**Table S2. The highly expressed circRNAs in GSE83521 microarray dataset***

| circRNAs ID | logFC | AveExpr | t | P.Value | adj.P.Val | B |
|---|---|---|---|---|---|---|
| hsa_circRNA_102614 | 5.114844 | 9.170761 | 5.358503 | 0.000151 | 0.018505 | 1.242062 |
| hsa_circRNA_103122 | 4.535221 | 8.875839 | 5.11926 | 0.000226 | 0.018647 | 0.856887 |
| hsa_circRNA_102191 | 4.067801 | 8.333783 | 5.961758 | 5.66E-05 | 0.010869 | 2.173576 |
| hsa_circRNA_100984 | 3.594655 | 9.460916 | 4.893295 | 0.000333 | 0.018647 | 0.485196 |
| hsa_circRNA_102777 | 3.416021 | 8.03436 | 4.598293 | 0.000558 | 0.02122 | -0.011 |
| hsa_circRNA_102615 | 3.383618 | 10.57047 | 4.36971 | 0.000838 | 0.026226 | -0.40336 |
| hsa_circRNA_102561 | 3.253066 | 7.223538 | 4.742967 | 0.000432 | 0.020407 | 0.23384 |
| hsa_circRNA_103559 | 3.218146 | 8.691229 | 4.328317 | 0.000903 | 0.026889 | -0.47509 |
| hsa_circRNA_102592 | 3.171836 | 8.528432 | 7.365163 | 7.02E-06 | 0.00539 | 4.118359 |
| hsa_circRNA_101471 | 3.07301 | 8.996952 | 5.737377 | 8.11E-05 | 0.012935 | 1.833834 |
| hsa_circRNA_104807 | 3.0546 | 8.03867 | 4.077322 | 0.001425 | 0.031787 | -0.91406 |
| hsa_circRNA_100385 | 2.860373 | 8.031314 | 4.302004 | 0.000947 | 0.027019 | -0.52079 |
| hsa_circRNA_102584 | 2.851011 | 7.359505 | 5.757389 | 7.85E-05 | 0.012935 | 1.86446 |
| hsa_circRNA_102815 | 2.787037 | 7.214228 | 4.759889 | 0.00042 | 0.020407 | 0.262291 |
| hsa_circRNA_104915 | 2.668114 | 7.635795 | 5.370022 | 0.000148 | 0.018505 | 1.260386 |
| hsa_circRNA_103594 | 2.661565 | 8.673063 | 4.401015 | 0.000792 | 0.025889 | -0.34925 |
| hsa_circRNA_104947 | 2.566432 | 7.781268 | 4.955062 | 0.000299 | 0.018647 | 0.587539 |
| hsa_circRNA_101309 | 2.553708 | 7.949005 | 4.717603 | 0.000452 | 0.020407 | 0.191119 |
| hsa_circRNA_102571 | 2.54568 | 7.563504 | 4.247619 | 0.001045 | 0.027977 | -0.61549 |
| hsa_circRNA_103783 | 2.394673 | 8.378795 | 3.624419 | 0.003294 | 0.049223 | -1.72005 |
| hsa_circRNA_100267 | 2.373285 | 7.046585 | 6.248964 | 3.62E-05 | 0.010093 | 2.596726 |
| hsa_circRNA_101308 | 2.344189 | 8.73575 | 2.900528 | 0.012879 | 0.101674 | -3.01941 |
| hsa_circRNA_103053 | 2.331664 | 7.503854 | 5.118023 | 0.000226 | 0.018647 | 0.854872 |
| hsa_circRNA_104589 | 2.045242 | 8.079576 | 8.682543 | 1.24E-06 | 0.001899 | 5.679028 |
| hsa_circRNA_102415 | 2.015402 | 9.631742 | 7.895387 | 3.41E-06 | 0.003487 | 4.775552 |
| hsa_circRNA_000684 | 1.961258 | 10.26201 | 4.766309 | 0.000415 | 0.020407 | 0.273076 |
| hsa_circRNA_101909 | 1.956629 | 6.496564 | 4.295015 | 0.000959 | 0.027019 | -0.53294 |
| hsa_circRNA_102564 | 1.90389 | 6.862762 | 4.248272 | 0.001044 | 0.027977 | -0.61435 |
| hsa_circRNA_102004 | 1.858887 | 7.237414 | 4.220126 | 0.001098 | 0.028582 | -0.66349 |
| hsa_circRNA_102082 | 1.811043 | 8.016018 | 2.466594 | 0.029036 | 0.151394 | -3.77911 |
| hsa_circRNA_104994 | 1.785076 | 7.260581 | 4.556818 | 0.0006 | 0.021429 | -0.0817 |
| hsa_circRNA_101267 | 1.7693 | 7.214811 | 4.104305 | 0.001356 | 0.031787 | -0.86656 |
| hsa_circRNA_101800 | 1.75497 | 7.608191 | 4.129601 | 0.001295 | 0.031561 | -0.82209 |
| hsa_circRNA_101882 | 1.687773 | 7.677626 | 11.02546 | 8.71E-08 | 0.000267 | 7.925097 |
| hsa_circRNA_100040 | 1.645425 | 7.637596 | 6.004156 | 5.30E-05 | 0.010869 | 2.236871 |
| hsa_circRNA_001653 | 1.640196 | 12.18023 | 2.698275 | 0.018844 | 0.121211 | -3.37706 |
| hsa_circRNA_400033 | 1.629349 | 11.33367 | 2.430888 | 0.031022 | 0.156433 | -3.84012 |
| hsa_circRNA_100493 | 1.578028 | 6.767506 | 4.955722 | 0.000299 | 0.018647 | 0.58863 |
| hsa_circRNA_104040 | 1.559607 | 8.312516 | 4.687329 | 0.000477 | 0.020632 | 0.140014 |
| hsa_circRNA_000200 | 1.533175 | 7.48334 | 2.394195 | 0.033198 | 0.160483 | -3.90252 |

* Selection criterion: $log_2FC \geqslant 1.5$, $P < 0.05$.

**Table S3. The highly expressed circRNAs in GSE93541 microarray dataset***

| circRNAs ID | logFC | AveExpr | t | P.Value | adj.P.Val | B |
|---|---|---|---|---|---|---|
| hsa_circRNA_104902 | 5.549918 | 6.395979 | 6.409715 | 0.000719 | 0.009633 | -0.14517 |
| hsa_circRNA_104081 | 5.379094 | 5.197306 | 17.24891 | 2.77E-06 | 0.000693 | 5.564941 |
| hsa_circRNA_100647 | 5.127569 | 5.844993 | 6.832409 | 0.000513 | 0.008468 | 0.225974 |
| hsa_circRNA_000082 | 5.056468 | 5.945115 | 15.15818 | 5.86E-06 | 0.000888 | 4.871626 |
| hsa_circRNA_100883 | 5.04177 | 9.082862 | 3.292856 | 0.016899 | 0.066209 | -3.6126 |
| hsa_circRNA_101522 | 5.022606 | 7.054242 | 5.453828 | 0.001655 | 0.015685 | -1.06337 |
| hsa_circRNA_001547 | 4.80241 | 5.639542 | 9.82981 | 6.96E-05 | 0.002937 | 2.378418 |
| hsa_circRNA_100560 | 4.762872 | 5.632243 | 6.575831 | 0.000628 | 0.009319 | 0.003076 |
| hsa_circRNA_104986 | 4.451131 | 5.761801 | 5.090469 | 0.002337 | 0.018623 | -1.44395 |
| hsa_circRNA_103814 | 4.434023 | 5.681234 | 12.42768 | 1.84E-05 | 0.001581 | 3.752427 |
| hsa_circRNA_104907 | 4.411472 | 6.16879 | 4.739302 | 0.003316 | 0.02341 | -1.82964 |
| hsa_circRNA_101704 | 4.395244 | 4.841887 | 12.5625 | 1.73E-05 | 0.001581 | 3.814544 |
| hsa_circRNA_400004 | 4.301286 | 5.152855 | 8.995348 | 0.000114 | 0.003639 | 1.851741 |
| hsa_circRNA_001459 | 4.26431 | 7.651127 | 10.54959 | 4.67E-05 | 0.002407 | 2.796221 |
| hsa_circRNA_100748 | 4.212109 | 5.611901 | 5.204993 | 0.002093 | 0.0175 | -1.32201 |
| hsa_circRNA_101412 | 4.040645 | 8.636371 | 14.78575 | 6.77E-06 | 0.000912 | 4.734631 |
| hsa_circRNA_102922 | 3.902368 | 5.945268 | 3.220858 | 0.018479 | 0.070956 | -3.7092 |
| hsa_circRNA_103002 | 3.770017 | 5.591002 | 3.919177 | 0.00803 | 0.041848 | -2.80226 |
| hsa_circRNA_100719 | 3.758885 | 7.201555 | 3.067254 | 0.022419 | 0.08094 | -3.91737 |
| hsa_circRNA_103212 | 3.757863 | 5.944571 | 5.078194 | 0.002365 | 0.018741 | -1.45713 |
| hsa_circRNA_104038 | 3.750359 | 5.576448 | 3.247928 | 0.017866 | 0.06952 | -3.67281 |
| hsa_circRNA_102415 | 3.710927 | 5.940576 | 3.663015 | 0.010807 | 0.050461 | -3.12689 |
| hsa_circRNA_101405 | 3.679184 | 5.934563 | 6.552603 | 0.00064 | 0.009319 | -0.01746 |
| hsa_circRNA_100040 | 3.524407 | 7.962445 | 2.528365 | 0.045328 | 0.131188 | -4.6651 |
| hsa_circRNA_104694 | 3.439457 | 6.196708 | 3.736609 | 0.009913 | 0.047739 | -3.03263 |
| hsa_circRNA_101839 | 3.432159 | 7.205079 | 2.577043 | 0.042471 | 0.126046 | -4.5968 |
| hsa_circRNA_101305 | 3.376097 | 6.7664 | 5.641945 | 0.001393 | 0.014186 | -0.87343 |
| hsa_circRNA_100115 | 3.3682 | 6.192718 | 2.830451 | 0.030404 | 0.100637 | -4.2432 |
| hsa_circRNA_105019 | 3.252407 | 6.251331 | 3.735645 | 0.009924 | 0.047739 | -3.03386 |
| hsa_circRNA_102082 | 3.24236 | 10.65089 | 18.95479 | 1.60E-06 | 0.000693 | 6.048886 |
| hsa_circRNA_101427 | 3.219474 | 6.557064 | 2.569878 | 0.042879 | 0.126827 | -4.60685 |
| hsa_circRNA_102379 | 3.217535 | 6.537761 | 2.9668 | 0.025487 | 0.087898 | -4.05493 |
| hsa_circRNA_400027 | 3.17686 | 9.874688 | 8.289287 | 0.00018 | 0.004916 | 1.366021 |
| hsa_circRNA_101404 | 3.161732 | 8.148588 | 5.304436 | 0.001904 | 0.016742 | -1.21762 |
| hsa_circRNA_102046 | 3.155135 | 7.212284 | 3.1167 | 0.021059 | 0.077792 | -3.85006 |
| hsa_circRNA_101753 | 3.111119 | 9.202917 | 6.495486 | 0.00067 | 0.009544 | -0.06823 |
| hsa_circRNA_104029 | 3.080117 | 7.235535 | 5.611234 | 0.001433 | 0.014419 | -0.90412 |
| hsa_circRNA_100879 | 3.058006 | 5.884435 | 3.614837 | 0.011441 | 0.052219 | -3.18902 |
| hsa_circRNA_104110 | 2.96896 | 7.195974 | 2.516925 | 0.046028 | 0.132558 | -4.68117 |
| hsa_circRNA_000855 | 2.869953 | 8.892158 | 13.8434 | 9.90E-06 | 0.001155 | 4.367214 |
| hsa_circRNA_100606 | 2.864535 | 6.175698 | 3.420949 | 0.014442 | 0.060498 | -3.4424 |

| | | | | | | |
|---|---|---|---|---|---|---|
| hsa_circRNA_104381 | 2.794117 | 6.041408 | 3.374345 | 0.015287 | 0.062477 | -3.50408 |
| hsa_circRNA_102113 | 2.747358 | 9.828945 | 6.972381 | 0.00046 | 0.008143 | 0.344562 |
| hsa_circRNA_104374 | 2.718753 | 7.109213 | 3.429679 | 0.01429 | 0.060147 | -3.43088 |
| hsa_circRNA_103727 | 2.690488 | 7.430931 | 2.583528 | 0.042105 | 0.125602 | -4.58771 |
| hsa_circRNA_103929 | 2.645205 | 7.851223 | 6.03847 | 0.000982 | 0.011505 | -0.4882 |
| hsa_circRNA_101945 | 2.606853 | 6.603082 | 2.456955 | 0.049892 | 0.139777 | -4.76541 |
| hsa_circRNA_101092 | 2.572469 | 7.439534 | 4.400894 | 0.00472 | 0.02941 | -2.21861 |
| hsa_circRNA_000200 | 2.561462 | 10.34396 | 10.91588 | 3.85E-05 | 0.002248 | 2.997103 |
| hsa_circRNA_102600 | 2.559545 | 8.66866 | 6.534364 | 0.00065 | 0.009324 | -0.03364 |
| hsa_circRNA_102445 | 2.525952 | 12.84816 | 7.347545 | 0.000347 | 0.007026 | 0.652389 |
| hsa_circRNA_103012 | 2.51223 | 7.13617 | 3.037901 | 0.023272 | 0.082823 | -3.95746 |
| hsa_circRNA_100799 | 2.474349 | 6.72164 | 3.309981 | 0.016545 | 0.065544 | -3.58973 |
| hsa_circRNA_103749 | 2.403072 | 9.640275 | 9.073915 | 0.000109 | 0.003571 | 1.903423 |
| hsa_circRNA_102441 | 2.387629 | 6.912386 | 3.448285 | 0.013971 | 0.059664 | -3.40636 |
| hsa_circRNA_104310 | 2.385998 | 12.74989 | 10.21541 | 5.61E-05 | 0.002649 | 2.606182 |
| hsa_circRNA_104050 | 2.374597 | 11.27209 | 11.01411 | 3.66E-05 | 0.002211 | 3.049702 |
| hsa_circRNA_102399 | 2.369437 | 6.953069 | 4.525015 | 0.004139 | 0.026831 | -2.07394 |
| hsa_circRNA_103723 | 2.348896 | 9.95965 | 4.821452 | 0.003051 | 0.022073 | -1.7378 |
| hsa_circRNA_104313 | 2.299962 | 8.672599 | 3.483551 | 0.013387 | 0.058166 | -3.36002 |
| hsa_circRNA_104635 | 2.297165 | 7.141666 | 4.340896 | 0.005033 | 0.03039 | -2.28938 |
| hsa_circRNA_101407 | 2.285528 | 8.52755 | 3.983108 | 0.007468 | 0.039726 | -2.72277 |
| hsa_circRNA_002086 | 2.281682 | 10.26546 | 7.192589 | 0.000389 | 0.007434 | 0.526978 |
| hsa_circRNA_102838 | 2.272879 | 11.90226 | 9.728673 | 7.38E-05 | 0.003004 | 2.317109 |
| hsa_circRNA_101943 | 2.247856 | 9.913325 | 5.235861 | 0.002032 | 0.017284 | -1.28945 |
| hsa_circRNA_100888 | 2.20019 | 6.624833 | 2.756098 | 0.033509 | 0.107104 | -4.34652 |
| hsa_circRNA_100104 | 2.160606 | 7.361002 | 9.30313 | 9.48E-05 | 0.003457 | 2.051645 |
| hsa_circRNA_102397 | 2.159984 | 7.640676 | 5.576219 | 0.001479 | 0.014716 | -0.93926 |
| hsa_circRNA_400040 | 2.136656 | 12.88406 | 7.942023 | 0.000227 | 0.005605 | 1.112159 |
| hsa_circRNA_101440 | 2.111057 | 7.082708 | 6.419524 | 0.000713 | 0.009633 | -0.13633 |
| hsa_circRNA_102810 | 2.102392 | 8.016356 | 6.76901 | 0.000539 | 0.008688 | 0.17157 |
| hsa_circRNA_104513 | 2.098685 | 9.905888 | 11.38645 | 3.03E-05 | 0.002041 | 3.244425 |
| hsa_circRNA_000881 | 2.083647 | 12.82592 | 6.744176 | 0.000549 | 0.008688 | 0.15014 |
| hsa_circRNA_101306 | 2.057336 | 7.418293 | 3.402454 | 0.014771 | 0.061289 | -3.46684 |
| hsa_circRNA_104099 | 2.051506 | 11.38357 | 5.126079 | 0.002258 | 0.018217 | -1.40583 |
| hsa_circRNA_000993 | 2.048863 | 10.10631 | 6.32799 | 0.000769 | 0.010052 | -0.21928 |
| hsa_circRNA_102631 | 2.039795 | 9.789093 | 8.587488 | 0.000148 | 0.004409 | 1.575959 |
| hsa_circRNA_000042 | 2.030713 | 9.477708 | 5.87801 | 0.001129 | 0.012205 | -0.64167 |
| hsa_circRNA_102442 | 2.025088 | 10.51807 | 6.478702 | 0.00068 | 0.009597 | -0.08322 |
| hsa_circRNA_100984 | 2.015618 | 6.147512 | 2.636617 | 0.039234 | 0.119695 | -4.51334 |
| hsa_circRNA_101887 | 1.98106 | 8.107677 | 3.076099 | 0.022169 | 0.080534 | -3.90531 |
| hsa_circRNA_100882 | 1.97699 | 9.771699 | 10.4427 | 4.95E-05 | 0.002463 | 2.736155 |
| hsa_circRNA_103085 | 1.96987 | 9.091516 | 2.466847 | 0.049232 | 0.138593 | -4.75151 |
| hsa_circRNA_102374 | 1.969544 | 10.47162 | 10.74827 | 4.21E-05 | 0.002361 | 2.90612 |

| | | | | | | |
|---|---|---|---|---|---|---|
| hsa_circRNA_102446 | 1.957439 | 9.064083 | 5.213382 | 0.002076 | 0.0175 | -1.31315 |
| hsa_circRNA_001040 | 1.946104 | 10.31297 | 8.34328 | 0.000173 | 0.004819 | 1.404573 |
| hsa_circRNA_100997 | 1.93874 | 8.407016 | 4.733866 | 0.003334 | 0.023446 | -1.83576 |
| hsa_circRNA_103801 | 1.922821 | 10.50599 | 5.437671 | 0.00168 | 0.015685 | -1.0799 |
| hsa_circRNA_104200 | 1.903873 | 8.170936 | 10.1695 | 5.75E-05 | 0.002649 | 2.57955 |
| hsa_circRNA_100891 | 1.890809 | 7.900134 | 4.295685 | 0.005285 | 0.031476 | -2.34307 |
| hsa_circRNA_001389 | 1.887989 | 9.553138 | 6.152423 | 0.000891 | 0.01099 | -0.38114 |
| hsa_circRNA_101373 | 1.866249 | 9.060177 | 9.055832 | 0.00011 | 0.003571 | 1.891568 |
| hsa_circRNA_002039 | 1.848158 | 7.409816 | 3.042865 | 0.023125 | 0.082469 | -3.95067 |
| hsa_circRNA_102385 | 1.846646 | 8.961994 | 6.10549 | 0.000928 | 0.011124 | -0.42504 |
| hsa_circRNA_103456 | 1.825122 | 7.376121 | 6.365996 | 0.000745 | 0.009814 | -0.18472 |
| hsa_circRNA_102728 | 1.823128 | 7.650097 | 5.421706 | 0.001705 | 0.015685 | -1.09628 |
| hsa_circRNA_102171 | 1.806955 | 7.624583 | 3.863973 | 0.008554 | 0.043794 | -2.87139 |
| hsa_circRNA_104864 | 1.805437 | 7.947371 | 7.151953 | 0.000401 | 0.007556 | 0.49369 |
| hsa_circRNA_100859 | 1.804105 | 6.697952 | 3.925658 | 0.007971 | 0.041789 | -2.79417 |
| hsa_circRNA_001264 | 1.799789 | 8.13657 | 5.152445 | 0.002201 | 0.0179 | -1.37773 |
| hsa_circRNA_104616 | 1.790622 | 9.233678 | 3.125772 | 0.020819 | 0.077397 | -3.83774 |
| hsa_circRNA_103137 | 1.78664 | 11.03265 | 7.491054 | 0.000312 | 0.00674 | 0.766429 |
| hsa_circRNA_104510 | 1.784374 | 8.471294 | 4.276394 | 0.005397 | 0.032032 | -2.36607 |
| hsa_circRNA_101030 | 1.782183 | 7.740915 | 4.346468 | 0.005003 | 0.030313 | -2.28278 |
| hsa_circRNA_101063 | 1.777336 | 6.871707 | 4.77353 | 0.003202 | 0.02298 | -1.79126 |
| hsa_circRNA_000911 | 1.768351 | 13.1492 | 7.655017 | 0.000278 | 0.006345 | 0.894306 |
| hsa_circRNA_000046 | 1.766822 | 9.317355 | 3.238793 | 0.01807 | 0.069973 | -3.68508 |
| hsa_circRNA_103608 | 1.752198 | 10.18141 | 5.750829 | 0.001264 | 0.013093 | -0.76564 |
| hsa_circRNA_000864 | 1.748896 | 12.12696 | 7.952063 | 0.000226 | 0.005605 | 1.119645 |
| hsa_circRNA_104401 | 1.743464 | 10.99593 | 6.545159 | 0.000644 | 0.009319 | -0.02406 |
| hsa_circRNA_104148 | 1.741904 | 7.869837 | 3.439677 | 0.014117 | 0.059864 | -3.4177 |
| hsa_circRNA_101136 | 1.739729 | 8.604194 | 6.83632 | 0.000511 | 0.008468 | 0.229317 |
| hsa_circRNA_100397 | 1.724886 | 8.180239 | 6.140527 | 0.0009 | 0.011024 | -0.39225 |
| hsa_circRNA_102041 | 1.721285 | 7.5571 | 3.814437 | 0.009056 | 0.044825 | -2.93382 |
| hsa_circRNA_100117 | 1.708388 | 8.764601 | 6.566486 | 0.000633 | 0.009319 | -0.00518 |
| hsa_circRNA_101248 | 1.701494 | 8.386806 | 4.661306 | 0.003592 | 0.024821 | -1.91777 |
| hsa_circRNA_102045 | 1.701277 | 8.809551 | 4.882579 | 0.002869 | 0.02102 | -1.67011 |
| hsa_circRNA_102551 | 1.690221 | 6.940558 | 3.819851 | 0.008999 | 0.044767 | -2.92698 |
| hsa_circRNA_100470 | 1.689887 | 8.138617 | 2.817272 | 0.030931 | 0.101804 | -4.26148 |
| hsa_circRNA_104803 | 1.66898 | 7.422129 | 2.972662 | 0.025296 | 0.087898 | -4.04687 |
| hsa_circRNA_100048 | 1.668478 | 7.501766 | 3.155445 | 0.020056 | 0.075479 | -3.79751 |
| hsa_circRNA_100832 | 1.663845 | 7.402915 | 4.547322 | 0.004043 | 0.026689 | -2.04819 |
| hsa_circRNA_103554 | 1.661797 | 9.150712 | 5.534251 | 0.001537 | 0.015036 | -0.98159 |
| hsa_circRNA_102924 | 1.659112 | 9.018112 | 6.427195 | 0.000709 | 0.009633 | -0.12942 |
| hsa_circRNA_001405 | 1.645692 | 11.30917 | 5.729694 | 0.001288 | 0.013264 | -0.78644 |
| hsa_circRNA_100422 | 1.644272 | 9.714228 | 8.530783 | 0.000153 | 0.004409 | 1.536592 |
| hsa_circRNA_103555 | 1.643711 | 12.24415 | 5.441088 | 0.001675 | 0.015685 | -1.0764 |

| | | | | | | |
|---|---|---|---|---|---|---|
| hsa_circRNA_000684 | 1.623346 | 10.10367 | 4.778571 | 0.003186 | 0.022957 | -1.78562 |
| hsa_circRNA_103134 | 1.601497 | 10.64736 | 4.602007 | 0.003819 | 0.026061 | -1.98538 |
| hsa_circRNA_001241 | 1.591908 | 10.56678 | 4.936834 | 0.002718 | 0.020427 | -1.61048 |
| hsa_circRNA_101861 | 1.586486 | 8.50022 | 4.937019 | 0.002718 | 0.020427 | -1.61028 |
| hsa_circRNA_100446 | 1.567078 | 7.335682 | 4.437371 | 0.00454 | 0.028906 | -2.17585 |
| hsa_circRNA_104400 | 1.546802 | 10.93791 | 8.206657 | 0.00019 | 0.005115 | 1.306551 |
| hsa_circRNA_104139 | 1.546641 | 6.736447 | 3.296612 | 0.01682 | 0.066185 | -3.60758 |
| hsa_circRNA_100147 | 1.540636 | 8.714112 | 4.40979 | 0.004675 | 0.029236 | -2.20817 |
| hsa_circRNA_001654 | 1.536069 | 10.97618 | 7.460572 | 0.000319 | 0.00674 | 0.742373 |
| hsa_circRNA_104126 | 1.522054 | 10.49393 | 6.912142 | 0.000482 | 0.008354 | 0.293782 |

* Selection criterion: $log_2FC \geqslant 1.5$, $P < 0.05$.

**Table S4. The potential circRERE(4-5)-binding miRNAs predicted by circBank database**

| miRNA | score | energy | alignLeft |
|---|---|---|---|
| hsa-miR-7974 | 168 | -27.27 | miRNA:3' cccGAGTC--CTCTCGTAGTGTCGGa 5' |
| hsa-miR-1270 | 163 | -17.49 | miRNA:3' tgtGTC--GAGAAGGTATAGAGGTc 5' |
| hsa-miR-3192-5p | 162 | -23.43 | miRNA:3' aaggTGACGATGT-TGGAGGGTCt 5' |
| hsa-miR-4731-5p | 161 | -26.530001 | miRNA:3' gtgtGAGTACAC-CGGGGGTCGt 5' |
| hsa-miR-571 | 159 | -18.26 | miRNA:3' gagtgaGTCTACCGGTTGAGt 5' |
| hsa-miR-6749-5p | 158 | -34.889999 | miRNA:3' cgaGGGGGTTG-GGGTCCGGGCt 5' |
| hsa-miR-6165 | 156 | -21.58 | miRNA:3' gagGGGAGTGGAGGACGAc 5' |
| hsa-miR-1207-5p | 154 | -37.779999 | miRNA:3' ggGGAGGGTCG---GAGGGACGGt 5' |
| hsa-miR-4433a-3p | 154 | -24.35 | miRNA:3' taCAGGGT---GGGGGTGAGGACa 5' |
| hsa-miR-6856-5p | 153 | -30.209999 | miRNA:3' ggTGTC-GTGGTGACGAGGAGAGAa 5' |
| hsa-miR-4763-3p | 152 | -36.27 | miRNA:3' ggGCGGGTCGTGGTCGGGGACGGa 5' |
| hsa-miR-4675 | 151 | -27.73 | miRNA:3' ggACGACCAGT-TAGTGTCGGgg 5' |
| hsa-miR-4722-5p | 151 | -27.09 | miRNA:3' gtTGGACCGTGTC----GG--GAGGACGg 5' |
| hsa-miR-4776-5p | 151 | -23.059999 | miRNA:3' tcggGAACGGTAGGACCAGGTg 5' |
| hsa-miR-892c-5p | 151 | -19.5 | miRNA:3' actgacCGTGGAAAGACTTat 5' |
| hsa-miR-6862-5p | 151 | -23.360001 | miRNA:3' tttcagaGAGGGTCGTACGGGc 5' |
| hsa-miR-892c-5p | 151 | -18.26 | miRNA:3' actgacCGTGGAAAGACTTat 5' |
| hsa-miR-4772-3p | 150 | -19.92 | miRNA:3' agactaGTCCGTTTCAACGTCc 5' |
| hsa-miR-6857-5p | 150 | -26.01 | miRNA:3' tgaCCGGACTGGGT-TAGGGGTt 5' |
| hsa-miR-6878-3p | 150 | -19.219999 | miRNA:3' gatcctCTTTC-TTCTCCGGTc 5' |
| hsa-miR-6892-3p | 150 | -25.09 | miRNA:3' gacgttccccaCCCTCTCCCt 5' |
| hsa-miR-6878-3p | 150 | -19.219999 | miRNA:3' gatcctCTTTC-TTCTCCGGTc 5' |
| hsa-miR-6892-3p | 150 | -25.09 | miRNA:3' gacgttccccaCCCTCTCCCt 5' |
| hsa-miR-4491 | 149 | -19.16 | miRNA:3' aaaccAGTGTGGTCAGGTGTaa 5' |
| hsa-miR-6800-3p | 149 | -27.629999 | miRNA:3' ccccGCTACGGTCCTCTCCac 5' |
| hsa-miR-6800-3p | 149 | -27.629999 | miRNA:3' ccccGCTACGGTCCTCTCCac 5' |
| hsa-miR-6843-3p | 148 | -21.66 | miRNA:3' gacgtCTCTTGTCCTCTGGTa 5' |
| hsa-miR-8060 | 148 | -25.5 | miRNA:3' caGGAGGATGGGTGACGAAGTACc 5' |
| hsa-miR-6843-3p | 148 | -21.66 | miRNA:3' gacgtCTCTTGTCCTCTGGTa 5' |
| hsa-miR-328-3p | 147 | -25.790001 | miRNA:3' tgcCTTCCCGTCTCTCCCGGtc 5' |
| hsa-miR-6730-5p | 147 | -22 | miRNA:3' agACTGTTGG-GGAG--GTGGAAAGa 5' |
| hsa-miR-328-3p | 147 | -25.790001 | miRNA:3' tgcCTTCCCGTCTCTCCCGGtc 5' |
| hsa-miR-637 | 146 | -22.98 | miRNA:3' tgcgtctcggGCTTTCGGGGGTCa 5' |
| hsa-miR-661 | 146 | -21.610001 | miRNA:3' tgcgcGTCCGGT-CTCTGGGTCCGt 5' |
| hsa-miR-4431 | 146 | -15.05 | miRNA:3' tgGA-AGATCAAAAG-TCTCAGCg 5' |
| hsa-miR-6803-5p | 146 | -28.110001 | miRNA:3' tgcgGGTCGGGGGGTGGGGGTc 5' |
| hsa-miR-4431 | 146 | -15.05 | miRNA:3' tgGA-AGATCAAAAG-TCTCAGCg 5' |
| hsa-miR-4683 | 145 | -19 | miRNA:3' taGCCCGCTCGTGACCTAGAGGt 5' |
| hsa-miR-3941 | 145 | -16.75 | miRNA:3' atactaggagtcaACACACATt 5' |
| hsa-miR-301a-5p | 144 | -20.549999 | miRNA:3' tcatCACGT-TA--TTTCAGTCTCg 5' |
| hsa-miR-3183 | 144 | -25.549999 | miRNA:3' aggctcGCTGAGGCTCTCTCCg 5' |

| | | | |
|---|---|---|---|
| hsa-miR-4726-5p | 144 | -24.59 | miRNA:3' ggTGAGGTCCGAGG--AGACCGGGa 5' |
| hsa-miR-4800-5p | 144 | -20.32 | miRNA:3' agGAAGGAAGGAGCCAGGTGa 5' |
| hsa-miR-6852-5p | 144 | -21.290001 | miRNA:3' gtaCAGGAGTCTTGGGGTCCc 5' |
| hsa-miR-6887-5p | 144 | -28.73 | miRNA:3' acAGGA-GAGGTAGACAGGGGGGt 5' |
| hsa-miR-301a-5p | 144 | -20.549999 | miRNA:3' tcatCACGT-TA--TTTCAGTCTCg 5' |
| hsa-miR-3183 | 144 | -25.549999 | miRNA:3' aggctcGCTGAGGCTCTCTCCg 5' |
| hsa-miR-1295b-3p | 143 | -22.35 | miRNA:3' aaCGGGTCT--AGGCACCGGATAa 5' |
| hsa-miR-6871-5p | 143 | -21.139999 | miRNA:3' cgTTGG-TGGGGCTTGAGGGTac 5' |
| hsa-miR-7110-3p | 142 | -21.610001 | miRNA:3' gacgtccCTTC-ACCCTCTCTCt 5' |
| hsa-miR-2114-5p | 142 | -17.93 | miRNA:3' ctggcgaagttcCTTCCCTGAt 5' |
| hsa-miR-6883-3p | 142 | -21.530001 | miRNA:3' gacTCCTCTCAC-TCTATCCCTt 5' |
| hsa-miR-7110-3p | 142 | -21.610001 | miRNA:3' gacgtccCTTC-ACCCTCTCTCt 5' |
| hsa-miR-4715-3p | 141 | -17.360001 | miRNA:3' taacCGACGTCAATTCCACCGTg 5' |
| hsa-miR-6504-5p | 141 | -17.469999 | miRNA:3' gacgTAATGTCGTGTCGGTct 5' |
| hsa-miR-7151-3p | 141 | -22.299999 | miRNA:3' actcgggtaaggTCGGACATc 5' |
| hsa-miR-3085-5p | 141 | -21.51 | miRNA:3' tgaggacCGGGAGTC--TTACCGTGGa 5' |
| hsa-miR-7151-3p | 141 | -22.299999 | miRNA:3' actcgggtaaggTCGGACATc 5' |
| hsa-miR-1910-3p | 140 | -15.99 | miRNA:3' acaGTAGGACGA-AGACGGag 5' |
| hsa-miR-4757-5p | 140 | -23.6 | miRNA:3' tgTGGCACTGCAGTGTCTCCGGa 5' |
| hsa-miR-4789-3p | 140 | -23.309999 | miRNA:3' atataTGTGGACGATACACAc 5' |
| hsa-miR-6501-3p | 140 | -16.51 | miRNA:3' tgACAAT--GGCGTCCGACGAGAcc 5' |
| hsa-miR-6510-5p | 140 | -18.629999 | miRNA:3' ctgaggAGAGAGAGGGGACGAc 5' |
| hsa-miR-6808-5p | 140 | -18.700001 | miRNA:3' gtaCCAG-GGTGGAGGGACGGAc 5' |
| hsa-miR-7109-5p | 140 | -21.950001 | miRNA:3' tcgtcccagaggaGGGGGGTc 5' |
| hsa-miR-8070 | 140 | -18.860001 | miRNA:3' acCTCAGTCGGCAGTTAGTGTa 5' |
| hsa-miR-10394-5p | 140 | -23.75 | miRNA:3' taccgcaagtggTCCTGGACGTCt 5' |
| hsa-miR-4757-5p | 140 | -23.6 | miRNA:3' tgTGGCACTGCAGTGTCTCCGGa 5' |
| hsa-miR-4782-5p | 140 | -16.139999 | miRNA:3' aaCTAACAGAAGTATAGGTCTt 5' |
| hsa-miR-5011-5p | 140 | -17.389999 | miRNA:3' ctcACGTACC-G--ACATATATAt 5' |
| hsa-miR-10394-5p | 140 | -23.75 | miRNA:3' taccgcaagtggTCCTGGACGTCt 5' |

**Table S5. The potential circRERE(4-5)-binding miRNAs predicted by circInteractome database**

| Mirbase ID | Site Type | context+ score | context+ score percentile |
|---|---|---|---|
| hsa-miR-1264 | 7mer-m8 | -0.103 | 90 |
| hsa-miR-330-3p | 7mer-m8 | -0.039 | 77 |
| hsa-miR-377 | 7mer-m8 | -0.108 | 87 |
| hsa-miR-571 | 7mer-m8 | -0.218 | 94 |
| hsa-miR-1270 | 8mer-1a | -0.242 | 97 |
| hsa-miR-620 | 8mer-1a | -0.242 | 97 |
| hsa-miR-637 | 7mer-m8 | -0.194 | 90 |
| hsa-miR-661 | 7mer-m8 | -0.267 | 97 |
