# Supplementary material for: Silencing of circRERE(4-5) inhibits ONECUT2-mediated tumorigenesis and metastasis in gastric cancer
Source: Front Immunol. 2026 Mar 6;17:1686702. doi: 10.3389/fimmu.2026.1686702 (PMC13002853; doi:10.3389/fimmu.2026.1686702)

The original uncropped western blots of Figures 4G, 4H, 4J, and 5A.

Figure 4G

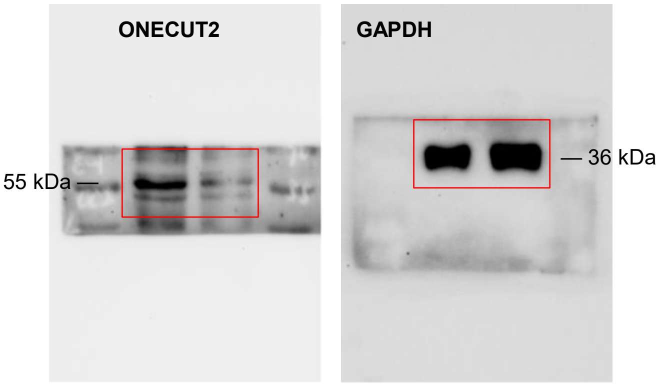

Figure 4H

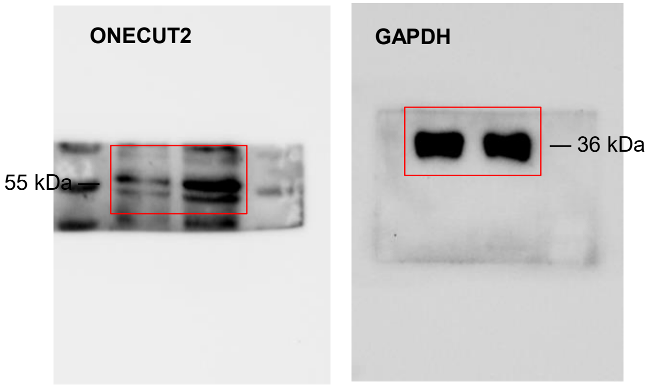

Figure 4J

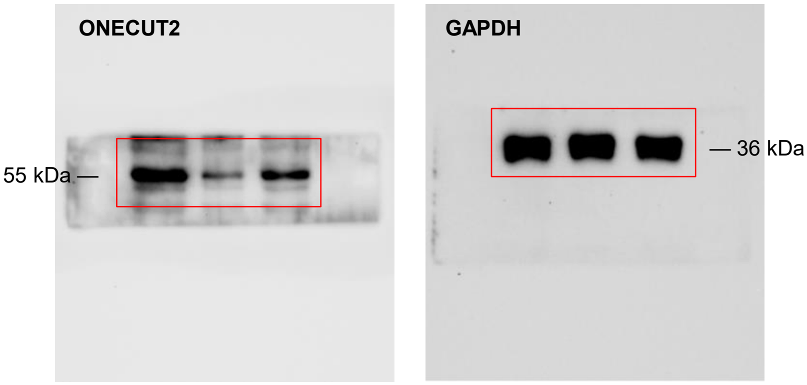

Figure 5A

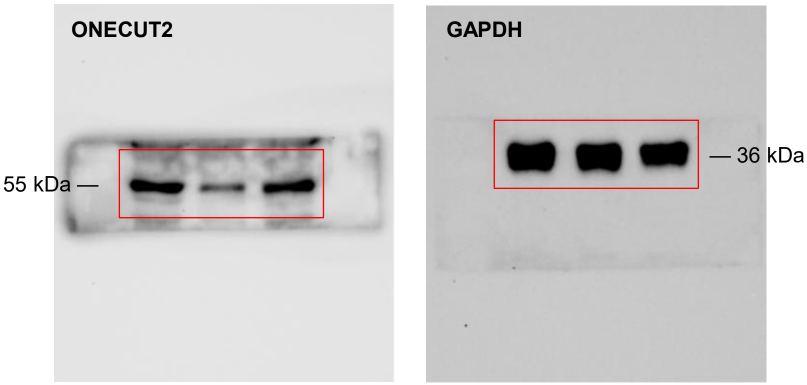

The original uncropped western blots of Figures S3E, S3F, S4A, S4G, and S5A.

Figure S3E

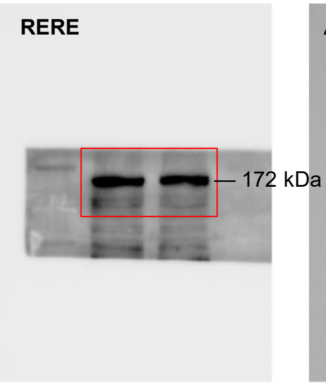

Figure S3F

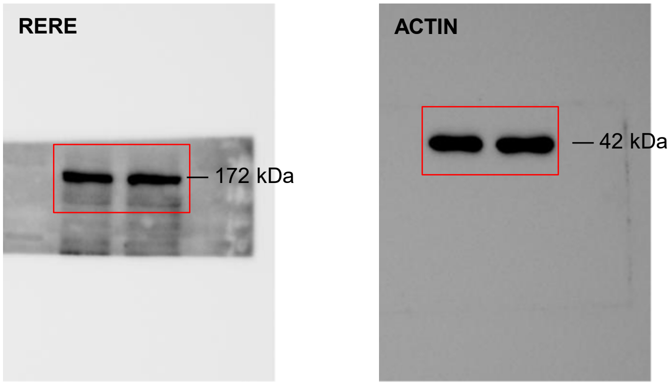

Figure S4A

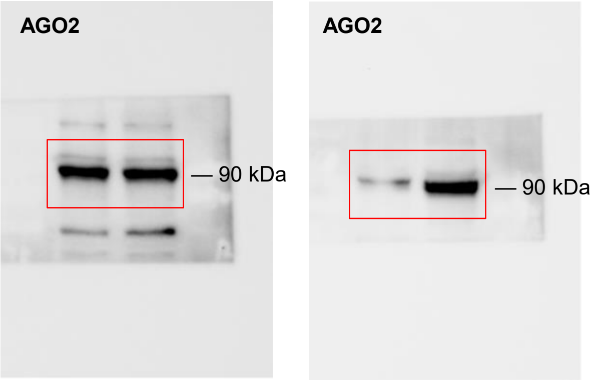

Figure S4G

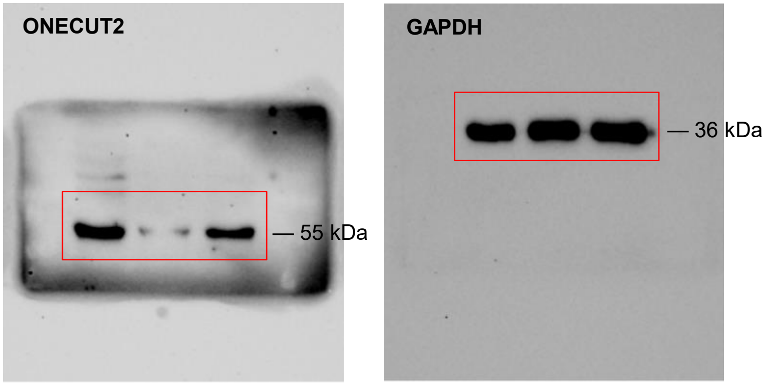

Figure S5A

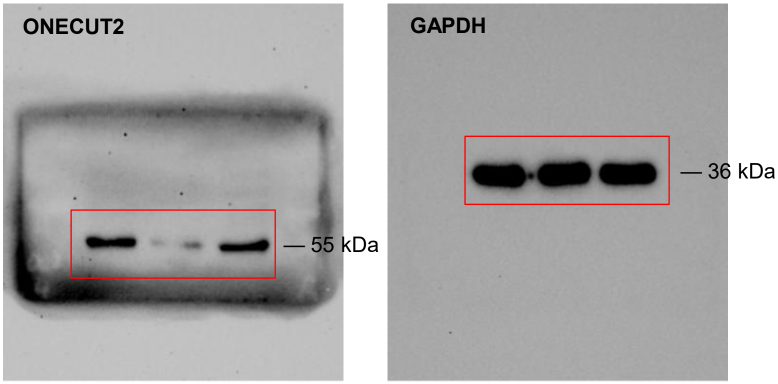

Supplement: Supplementary file 2 [file DataSheet2.pdf]
